# Pyruvate kinase, a metabolic sensor powering glycolysis, drives the metabolic control of DNA replication

**DOI:** 10.1186/s12915-022-01278-3

**Published:** 2022-04-13

**Authors:** Steff Horemans, Matthaios Pitoulias, Alexandria Holland, Emilie Pateau, Christophe Lechaplais, Dariy Ekaterina, Alain Perret, Panos Soultanas, Laurent Janniere

**Affiliations:** 1 Génomique Métabolique, Genoscope, Institut François Jacob, CEA, CNRS, Université Evry, Université Paris-Saclay, 91057 Evry, France; 2 Biodiscovery Institute, School of Chemistry, University of Nottingham, University Park, Nottingham, NG7 2RD UK

**Keywords:** Replication timing, Central carbon metabolism, Glycolytic enzymes, Replication enzymes, Cell cycle, Allosteric regulation, Protein phosphorylation, PEPut domain, Signaling

## Abstract

**Background:**

In all living organisms, DNA replication is exquisitely regulated in a wide range of growth conditions to achieve timely and accurate genome duplication prior to cell division. Failures in this regulation cause DNA damage with potentially disastrous consequences for cell viability and human health, including cancer. To cope with these threats, cells tightly control replication initiation using well-known mechanisms. They also couple DNA synthesis to nutrient richness and growth rate through a poorly understood process thought to involve central carbon metabolism. One such process may involve the cross-species conserved pyruvate kinase (PykA) which catalyzes the last reaction of glycolysis. Here we have investigated the role of PykA in regulating DNA replication in the model system *Bacillus subtilis.*

**Results:**

On analysing mutants of the catalytic (Cat) and C-terminal (PEPut) domains of *B. subtilis* PykA we found replication phenotypes in conditions where PykA is dispensable for growth. These phenotypes are independent from the effect of mutations on PykA catalytic activity and are not associated with significant changes in the metabolome. PEPut operates as a nutrient-dependent inhibitor of initiation while Cat acts as a stimulator of replication fork speed. Disruption of either PEPut or Cat replication function dramatically impacted the cell cycle and replication timing even in cells fully proficient in known replication control functions. In vitro, PykA modulates activities of enzymes essential for replication initiation and elongation via functional interactions. Additional experiments showed that PEPut regulates PykA activity and that Cat and PEPut determinants important for PykA catalytic activity regulation are also important for PykA-driven replication functions.

**Conclusions:**

We infer from our findings that PykA typifies a new family of cross-species replication control regulators that drive the metabolic control of replication through a mechanism involving regulatory determinants of PykA catalytic activity. As disruption of PykA replication functions causes dramatic replication defects, we suggest that dysfunctions in this new family of universal replication regulators may pave the path to genetic instability and carcinogenesis.

**Supplementary Information:**

The online version contains supplementary material available at 10.1186/s12915-022-01278-3.

## Background

Propagation of genomes between generations requires timely and accurate duplication of DNA by well-known (hereafter termed “classical”) regulatory mechanisms that control the activity of replication initiators and origins [1, 2]. However, for coordinating DNA synthesis with nutrient richness and growth rate, replication must also be under a metabolic control. This additional level of control operates by modulating the initiation and elongation phases of replication. In bacteria, the net result is a precise and reproducible timing of DNA synthesis in the cell cycle across a wide range of nutritional conditions and growth rates [3–6]. In eukaryotes, the metabolic control is thought to confine DNA synthesis to the reduction phase of a redox metabolic cycle, reiterated several times per cell cycle [7–13]. The mechanism of the metabolic control of replication remains largely unknown as well as its interface with “classical” replication control functions and importance for cell survival.

In bacteria, long-standing hypotheses postulating that the metabolic control of replication operates by modulating the concentration of replication precursors or the concentration of the active form of the replication initiator DnaA (DnaA-ATP), have been challenged [14–18]. It is also unlikely that this control operates by modulating the activity of DnaA and *oriC* regulators, as replication still responds to metabolism in cells lacking such regulators [15, 19, 20]. Hence, several groups have argued that the metabolic control of replication is a multifactorial process which senses nutrient richness and communicates this information to the replication machinery (see for instance [7, 9, 13, 21–24]. The guanosine tetra- and penta-phosphate that signals the cellular metabolic state and impacts replication initiation and elongation at basal concentrations may be one of these regulators [25–27] (see however [15, 28]).

Nutrient richness is exquisitely sensed by a metabolic area termed central carbon metabolism (CCM) [29, 30]. This metabolic area encompasses about 30 highly conserved enzymes and reactions that extract from nutrients the precursors and energy needed for macromolecular synthesis and biomass production. By directly sensing the supply and the demand in biosynthetic reactions, CCM may be at a strategic position for orchestrating the metabolic control of replication. This hypothesis is supported by an ever-increasing core of data uncovering CCM-replication links from bacteria to higher eukaryotes.

In *Escherichia coli*, several CCM-replication links connect the replication initiator DnaA to CCM metabolites. They may thus couple initiation to CCM activity. First, cyclic AMP, a metabolite that regulates CCM, interacts with DnaA to stimulate its binding to the replication origin and facilitate DnaA rejuvenation from the inactive DnaA-ADP to the active DnaA-ATP form [31]. Second, changes in the pyruvate-acetate node suppress initiation defects of a DnaA mutant (*dnaA46*) [32–34]. Third, two metabolites of the pyruvate-acetate node (acetyl-CoA and acetyl-phosphate) drive DnaA acetylation to prevent DnaA from binding ATP and *oriC* [35]. Other *E. coli* studies uncovered links that may couple elongation to metabolism [32, 33, 36]. In another proteobacteria (*Caulobacter crescentus*), the citrate synthase CitA which ensures the first reaction of the Krebs (tricarboxylic acid) cycle promotes initiation by negatively regulating the master driver of the cell cycle, CtrA [37].

**Fig. 1:**
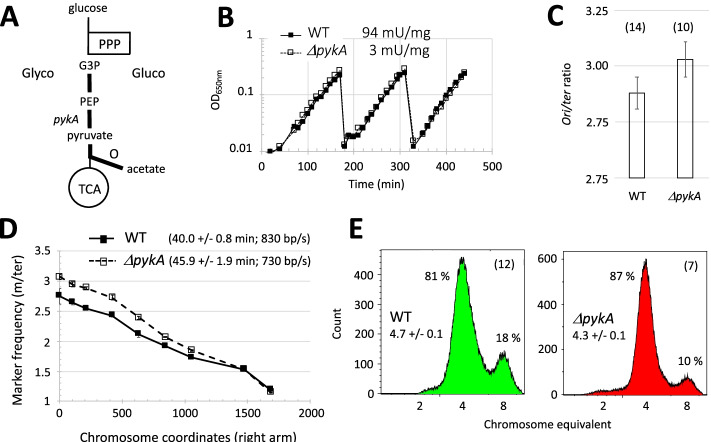
Replication analysis in wild-type (WT) and *pykA* null cells growing exponentially in MC medium. **A** Schematic representation of CCM. Glyco: Glycolysis; Gluco: gluconeogenesis; PPP: pentose phosphate pathway; TCA: tricarboxylic acid cycle; O: overflow pathway; G3P: glyceraldehyde 3-phosphate; PEP: phosphoenolpyruvate; Gray arrows: carbon flux; Thick bars: CCM area genetically linked to replication genes [42, 44]. **B** Growth rate and pyruvate kinase activity. Cells were grown exponentially for more than 20 generations using successive dilutions. Growth was assessed by spectrophotometry (OD_650nm_, a typical experiment is shown) and pyruvate kinase activity was determined in crude extracts (mean from six independent experiments). **C** *Ori/ter* ratio: *Ori/ter* ratios were determined by qPCR using as template total DNA extracted from growing cells. Numbers in brackets stand for the number of independent measurements. The Mann-Whitney *U* test showed that the ratios in wild-type and *ΔpykA* cells are significantly different at *p* < .05 (Table S1). **D** DNA elongation: Parameters of DNA elongation were determined using a marker frequency analysis by qPCR. The nearly monotonous decrease of marker frequencies from the origin to the terminus showed that there is no pause site along the chromosome (a typical experiment is shown). Numbers in brackets refer to C period (mean and SD from at least 3 experiments) and mean fork speed. **E** Number of origins per cell: To determine the number of origins/cell, chloramphenicol was added to exponentially growing cells. The drug inhibits replication initiation and cell division but allows completion of ongoing rounds of replication. After 4 h of drug treatment (runout experiment), cells were analyzed by flow cytometry after DNA staining. Panels: typical runout DNA histograms with the % of cells containing 4 and 8 chromosomes. Numbers in brackets stand for the number of independent reiterations of the experiment. The number of origins/cell (mean and SD) is given below the strain name. See “Methods” for details

CCM-replication links have also been discovered in the model Gram-positive bacterium *Bacillus subtilis*. First, subunits of the pyruvate dehydrogenase (PdhC) and related enzymes bind the origin region, the replication enzymes DnaC (helicase) and DnaG (primase) and/or inhibit replication initiation [38–41]. Second, CCM mutations suppress initiation and/or elongation defects in mutants of DnaC, DnaG, and the lagging-strand polymerase DnaE. They also disturb replication initiation and elongation in a medium-dependent manner [42–44]. Third, the metabolic control of replication is disturbed in cells mutated for PdhB, the glyceraldehyde 3-phosphate dehydrogenase (GapA), or the pyruvate kinase (PykA) [15]. Collectively, these results suggest that replication coupling to CCM activity in *B. subtilis* may depend on metabolic signals originating from the CCM area converting glyceraldehyde 3-phosphate to acetate (thick bars Fig. 1A) and on CCM-driven modulation of activities of replication enzymes involved in initiation and elongation (DnaC, DnaG, and DnaE) [43, 45–49]. Although the underlying mechanism remains elusive, it was proposed that in the rich LB medium, CCM modulates initiation by altering the functional recruitment of DnaC, DnaG, and DnaE to *oriC* and affects elongation by altering the activity of the lagging-strand polymerase DnaE in replication forks [44].

CCM-replication links were also found in eukaryotes ( [50–54] and see below). At first, these findings are surprising as metabolism and replication occur in different cellular compartments (cytoplasm and nucleus, respectively). However, data accumulating since the late 1950s show that this dogma has exceptions, with numerous CCM enzymes shown to be present in the nucleus for ensuring non-metabolic (moonlighting) functions (see for instance [55–62]. Hence, CCM-replication links in eukaryotes may involve nuclear CCM determinants. Several data support this notion. For instance, the timing of origin firing in eukaryotic cells depends on an increase in acetyl-CoA that promotes histone acetylation in the nucleus. This increase is geared by a metabolic cycle in yeast and by nuclear forms of the ATP-citrate lyase and Pdh complexes in mammalian cells [63–65]. In addition, the nuclear form of GAPDH and the lactate dehydrogenase are an integral part of a cofactor which, in association with the transcription factor Oct-1, stimulates S phase progression by inducing expression of histone H2B [66, 67]. In contrast, repression of histone genes by exogenous pyruvate delays S phase entry [68]. It was also found that a nuclear form of the phosphoglycerate kinase (PGK) interacts with the protein kinase CDC7 to positively regulate replication initiation by keeping the stimulatory effect of CDC7 on the MCM helicase [69]. Finally, some nuclear CCM enzymes (PGK, GAPDH and lactate dehydrogenase) modulate the activity of eukaryotic replicative polymerases (Polα, Polε, and Polδ) in vitro [70–72]. Collectively, these data suggest that CCM may be of prime importance for the metabolic control of replication from bacteria to human cells.

Here, we report on the relationship between PykA and DNA replication in *B. subtilis*. PykA is an ancestral protein that is highly conserved and abundant throughout the evolution tree. It catalyzes the irreversible transfer of phosphate from phosphoenolpyruvate (PEP) to ADP resulting in the production of pyruvic acid and ATP in the last reaction of glycolysis. The 3D structure of the functional homotetramer has been solved for many organisms [73]. It highlights the conservation of their global architecture and of their active site and enabled the identification of residues important for substrates (PEP and ADP) binding and for phosphoryl transfer in *B. subtilis* PykA (Fig. S1A). Almost all pyruvate kinases are homotropically activated by the substrate PEP. They are also allosterically regulated by heterotropic effectors. Crystal structures of the four PykA states (apo, substrate-complex, effector-complex, and substrate-effector-complex) show that the mechanism of PykA regulation involves conformational changes within each subunit and between neighboring subunits promoted by the binding of effectors. This suggests that cells harbor different conformers at levels depending on effector concentrations and typifying nutritional conditions, with the active R-state conformer at a concentration sufficient to fulfill cellular biosynthetic needs. The heterotropic effectors of *B. subtilis* PykA are AMP and ribose 5-phosphate [74]. They are predicted to stabilize the active conformer in a process involving an extra C-terminal domain (see below) [75]. Interestingly, *B. subtilis* PykA is constitutively expressed and produced in rather large amounts across a wide range of nutritional conditions, including gluconeogenic media where *pykA* is dispensable for growth [76, 77]. This suggests that PykA may have more than one function.

The PykA proteins of *B. subtilis* and related species have an extra C-terminal sequence (residues 477-585) [78, 79]. It interacts with the catalytic domain (termed thereafter Cat) through a hydrogen bond between E209 and L536 of neighboring subunits [74]. Interestingly, the sequence and 3D structure of the extra C-terminus are homologous to one of the PEP utilizer (PEPut) domains found in the EI component of the phosphoenolpyruvate:carbohydrate phosphotransferase system, the pyruvate phosphate dikinase (PPDK), and the PEP synthase (PEPS) (Fig. S1B) [74, 78]. Interestingly, a TSH motif mapping immediately downstream of the Cat-PEPut interacting L536 residue is conserved in PEPuts (Fig. S1B) [78]. In EI, the histidine residue of the motif is phosphorylated at the expense of PEP and the phosphoryl group is transferred to a protein (Hpr) during the process of sugar transport [80, 81]. In PPDK and PEPS, this H residue is a key part of the catalytic site: it is essential for the reversible conversion of pyruvate into PEP and the reaction depends on the transfer of a phosphoryl group from ATP or PEP to pyruvate or AMP through transient phosphorylation of the conserved histidine [82, 83]. The T residue of the TSH motif is phosphorylated for inhibiting the catalytic activity of H in PEPS and PPDK and its phosphorylation/dephosphorylation is catalyzed by a serine/threonine kinases of the DUF299 family using ADP as donor [84–87]. The function of the S residue of the TSH motif is unknown [84]. This residue is however phosphorylated in *B. subtilis* and *Arabidopsis thaliana* [88–90]. As the Cat-PEPut interaction stabilized by a hydrogen bond between E209 and L536 is predicted to assist heterotrophic effectors in regulating PykA activity [75] and as L536 is in immediate proximity with the TSH motif, posttranslational modifications of the motif may contribute to PykA regulation by modulating the Cat-PEPut interaction. This hypothesis is consistent with studies showing that posttranslational modifications, which are prevalent from bacteria to eukaryotes, affect CCM enzyme activities through different mechanisms and help the cell to respond to nutrient availability and fluctuations [91, 92].

Here, we demonstrate that PykA is a medium-dependent effector of replication with PEPut operating as an inhibitor of initiation and Cat, as a stimulator of elongation. These functions depend neither on PykA catalytic activity nor on changes in the metabolome measurable by LC/MS. In contrast, they may depend on functional interactions of PykA with the helicase DnaC, primase DnaG, and lagging-strand polymerase DnaE. Surprisingly, the PEPut and Cat replication functions are of prime importance for replication timing even in the presence of “classical” replication control functions. Finally, we found that PEPut regulates PykA catalytic activity and that PykA allosteric regulation and PykA replication functions share requirements in substrate binding to Cat, the Cat-PEPut interaction, and the TSH motif and its phosphorylation status. We propose from these findings that cells use the metabolic sensor capacity of PykA for integrating metabolism with DNA replication and that PykA typifies a new family of regulators gearing the metabolic control of replication. An intertwining regulatory model is discussed.

## Results

### Replication defects in pykA null cells occur in a medium where PykA is dispensable for growth

Replication phenotypes in *ΔpykA* cells were found in glycolytic regimens where PykA depletion causes a dramatic reduction in cellular metabolism and growth rate [15, 42, 44]. Here, we showed that a mere decrease in metabolism and/or growth rate is not mandatory for replication phenotypes to occur as replication defects in *ΔpykA* cells still happen in a gluconeogenic medium where PykA is dispensable for growth (MC medium, Fig. 1B). In this medium, runout flow cytometry studies showed a lower number of origin sequences per cell in the mutant in comparison to the wild-type strain, while qPCR studies showed that the mutant has a higher *ori/ter* ratio (the relative copy number of origin sequences versus terminus sequences), an extended C period (the time required for replicating the entire chromosome), and a lower speed of replication forks (Fig. 1C–E and Table S1). It is inferred from this that a moderate metabolic change ensuing depletion of PykA catalytic activity (this activity is reduced 30-fold in the mutant compared to the wild-type strain (Fig. 1B)) and/or the absence of the PykA protein, suffices for replication defects to occur in *ΔpykA* cells.

### Cat and PEPut affect replication independently of their metabolic functions

#### *Ori/ter* ratio analysis in Cat mutants

To further investigate the relationship between PykA and replication, DNA synthesis was analyzed in PykA catalytic mutants grown in MC. The mutants used were either deleted for the catalytic domain (*pykA*_*Δcat*_) or contained point mutations impeding either PEP binding, ADP binding (*pykA*_*R32A*_, *pykA*_*R73A*_*, pykA*_*GD245-6AA*_, and *pykA*_*T278A*_), or the phosphoryl transfer during catalysis (*pykA*_*K220A*_) (Fig. S1A) [73, 93]. We also used a strain containing a 27-amino-acid deletion (residues 208-234; *pykA*_*JP*_) encompassing residues involved in the stabilization of the Cat-PEPut interaction (E209) and in the phosphoryl transfer (K220). This deletion is a spontaneous suppressor of thermosensitive DnaE mutations [42]. Replication was assessed using the *ori/ter* ratio, a parameter sensitive to initiation and elongation defects and convenient for screening a rather large number of strains.

**Fig. 2:**
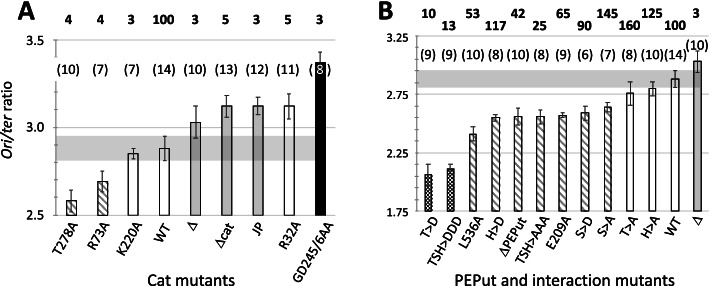
*Ori/ter* ratios and PykA catalytic activities in Cat and PEPut mutants. *Ori/ter* ratios were grouped (color code) according to the Mann-Whitney *U* test at a significance of *p* < 0.01 (see Table S1 for details). Numbers in brackets refer to the number of independent iterations. The horizontal gray bars highlight the wild-type ratio area. Pyruvate kinase activities (bolded numbers expressed in % of wild-type activity) in crude extracts were determined from at least three independent experiments (SD/means < 10%). **A** Cat mutant analysis. **B** PEPut mutant analysis. See Fig. 1 for details

Four classes of mutants characterized by a low, wild-type, high and notably high ratio were identified using the Mann-Whitney statistical *U* test run at a significance *p* < 0.01 (Fig. 2A, Table S1). Control experiments showed that the tested mutations dramatically inhibit (or abolish) PykA metabolic activity (Fig. 2A) and do not affect growth in MC (Fig. S2, left panel). Hence, replication defects in Cat mutants occur in the absence of a mere decrease in growth. Residues important for a proper *ori/ter* ratio are R32, R73, T278, GD245/6, and those deleted in PykAJP (208-234). Since mutants display different *ori/ter* ratios and similar residual pyruvate kinase activities, no obvious relationship links PykA catalytic activity to replication.

#### *Ori/ter* ratio analysis in PEPut mutants

Next, we analyzed the *ori/ter* ratio in a collection of PEPut mutants comprising a strain encoding a PykA protein deleted for PEPut (*pykA*_*ΔPEPut*_) and mutants of the TSH motif in which individual amino acids, or the whole motif, were replaced by A or D, the latter residue mimicking phosphorylation. We also tested mutants of the Cat-PEPut interaction (*pykA*_*E209A*_ and *pykA*_*L536A*_).

Three classes of PEPut mutants characterized by a very low, low, and wild-type ratio were identified by the Mann-Whitney *U* test run at a significance *p* < 0.01 (Fig. 2B, Table S1). Residues important for a proper ratio are the S residue of the TSH motif and the amino acids stabilizing the Cat-PEPut interaction (E209 and L536), as corresponding A mutants have a low ratio. The T and H residues of the TSH motif may also be important for replication in conditions favoring their phosphorylation, as the corresponding D mutants are associated with a very low and low ratio, respectively (Fig. 2B). Interestingly, PEPut mutations notably affect (positively and negatively) PykA catalytic activity (Fig. 2B) and do not alter growth in MC (Fig. S2, right panel). Moreover, PykA activity and *ori/ter* ratio do not covary (Fig. 2B).

We inferred from these findings that Cat and PEPut are endowed with non-metabolic functions allowing PykA to modulate DNA replication independently of its catalytic activity, and that the engagement of PEPut in replication may depend on posttranslational modifications of the TSH motif. Our results also show that PEPut is a new, potent regulator of PykA catalytic activity.

### Cat and PEPut mutations do not affect the metabolome in MC

**Fig. 3:**
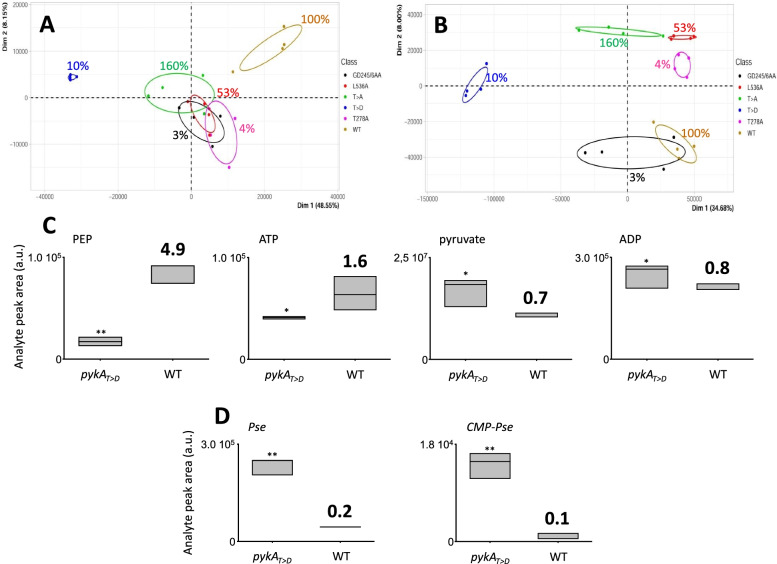
Metabolome analysis of wild-type and pykA mutants in MC. **A, B** PCA scores plots (first two components) in the positive (**A**) and negative (**B**) ionization mode. Ellipses are the 95% confidence regions. Indicated are the relative activities of PykA. Data correspond to 4 independent extractions (liquid cultures). **C** Comparison of PykA substrate and product contents in *pykA*_*T>D*_ and wild-type (WT) cells. **D** Comparison of the contents of compounds annotated as pseudaminic acid (Pse) and CMP-pseudaminic acid (CMP-Pse) in *pykA*_*T>D*_ and wild-type cells. Data in **C** and **D** correspond to 3 independent extractions (solid cultures).*, *p* > 0.05 ; **, *p* < 0.01 (Welch’s *T*-test). Values in bold panels **C** and **D** indicate the fold change for each metabolite (wild-type versus *pykA*_*T>D*_)

To better characterize the effect of PykA mutations on cellular metabolism in MC, the metabolome of cells hosting a PykA catalytic activity ranging from 3 to 160% (the wild-type strain and mutants *pykA*_*GD245/6AA*_, *pykA*_*T278A*_, *pykA*_*T>D*_, *pykA*_*L536A*_, and *pykA*_*T>A*_ which have a PykA activity 3, 4, 10, 53, and 160% of wild-type cells, respectively) was analyzed. For each strain, metabolomes were prepared from 4 independent cultures and analyzed by LC/MS. Untargeted analysis was carried out using XCMS and yielded 1960 and 14335 features in the positive and negative ionization modes, respectively, with automatic compound annotation (Kegg database; Table S2). Principal components analysis (PCA) was conducted on the full data matrix. The PCA score plots (Fig. 3A, B) showed that Dimension 1 capture most of the variance in both modes. In the positive mode, this axis clearly separates the wild-type and *pykA*_*T>D*_ samples while the remaining data are mixed into each other. In the negative mode, *pykA*_*GD245/6AA*_, *pykA*_*T278A*_, *pykA*_*L536A*_, and *pykA*_*T>A*_ are better segregated along Dimension 2, while wild-type and *pykA*_*T>D*_ remain well separated in Dimension 1, with *pykA*_*T>D*_ samples clearly away from the others. A closer inspection of PCA loadings plots identified 14 variables having the largest effect on each component. Of these, six have a biased signal due to poor correction of retention times and three are not significantly affected (fold change <3) (Stuani et al. 2014) as compared to the wild-type strain. Remaining variables pointed to molecules that do not correspond to any compound of the Kegg database or to molecules that are not expected to participate in *B. subtilis* metabolism. Altogether, this analysis did not detect significant changes in metabolite (including detectable nucleotides) concentrations and uncovered no relationship between score plots and PykA activity.

To further characterized the effect of PykA mutation on the metabolome, a study based on a procedure allowing detection of metabolites with short lifetime was carried out on the wild-type and *pykA*_*T>D*_ strains (see Experimental Procedures) [94]. This analysis confirmed the inference of Fig. 3A,B and the lack of significant changes in NTP pools (Fig. S3). A targeted analysis on PykA substrates and products revealed no significant change in ATP, pyruvate, and ADP concentrations and a 4.9 lower PEP content in the mutant (Fig. 3C). The data also uncovered an ion that accumulated in the *pykA*_*T>D*_ mutant, annotated as pseudaminic acid (Pse) in the Kegg database (Fig. 3D). Consistently, another accumulating ion annotated as a derivative of Pse (CMP-Pse) was found in the mutant. Putative Pse was observed mainly in the negative mode at m/z 333.1296 but was also detected in the positive mode at m/z 335.1443 (Fig. S4A, B). These two m/z values were anticipated to be the deprotonated [M−H]^−^ and protonated [M+H]^+^ forms of the molecule, respectively. Fragmentation pattern and hydrogen/deuterium exchanges (HDX) were also fully consistent with the Pse structure. Pse harbors 6 mobile protons. We determined the mobile proton number of m/z 333 in positive and negative ionization modes using in-solution HDX approach. Mobile protons (7 and 5) were observed in the positive and negative mode, respectively; i.e., 6 mobile protons for the neutral form (Fig. S4C-F). However, data were also in agreement with a previous study which deciphered the biosynthetic pathway of a Pse isomer: legionaminic acid (Leg) [95]. As this isomer should display the same fragmentation patterns under collision-induced dissociation (CID) as Pse, we considered m/z 333 in mutants as Leg. The signal was indeed absent in cells deleted for the Leg synthase (*ΔspsE*), confirming m/z 333 as Leg. Moreover, Leg accumulated in cells inactivated for the enzyme converting Leg into CMP-Leg (CMP-Leg synthase; *ΔspsF*) and the CMP-Leg signal was detected in wild-type and *pykA*_*T>D*_ cells but not in mutants of the Leg pathway (Fig. S4G, H). Finally, PEP depletion in *pykA*_*T>D*_ likely result from Leg production as PEP is a precursor of Leg [95] and as metabolome data suggested that PEP concentration inversely correlates with Leg concentration (Fig. 3C, D).

Changes in Leg, CMP-Leg and PEP concentration prompted us to analyze replication in Leg pathway mutants (*ΔspsE*, *ΔspsF*, and *ΔspsABCDEF*) in wild-type and *pykA*_*T>D*_ contexts. Results showed that neither the depletion of Leg, CMP-Leg, and/or PEP nor the accumulation of Leg affect the *ori/ter* ratio in both contexts showing that the Leg pathway is not involved in replication (ratios in single and double mutants vary by less than 5% compared to the wild-type and *pykA*_*T>D*_ parental strains). Collectively, our findings show that PykA mutations do not notably disturb the cellular metabolome (including nucleotide pools) in MC. Some mutants may however significantly (>3-fold) impact PEP, Leg, and CMP-Leg concentration but these changes are not responsible for the replication phenotypes.

### PEPut and Cat affect initiation and elongation, respectively

Changes in *ori/ter* ratio may result from changes in initiation and/or elongation. To determine which replication step is primarily defective in PykA mutants, cells replicating the chromosome from a plasmid replicon rather than from the cellular DnaA/*oriC* initiation complex were used. As argued previously [15, 44], if replication defects in *pykA* mutants were to result from changes in initiation, cells replicating their genome from the plasmid replicon would not suffer from metabolic mutation and would thus exhibit the *ori/ter* ratio of PykA+ *oriN*-dependent cells. In contrast, if replication defects were to result from changes in elongation, plasmid replicon-dependent cells would still suffer from the *pykA* mutation and would thus have an *ori/ter* ratio different from PykA+ *oriN*-dependent cells. The analysis was carried out in the *ΔoriC* context rather than in the *ΔdnaA* context to avoid any interference of DnaA depletion on genome expression [96].

**Fig. 4:**
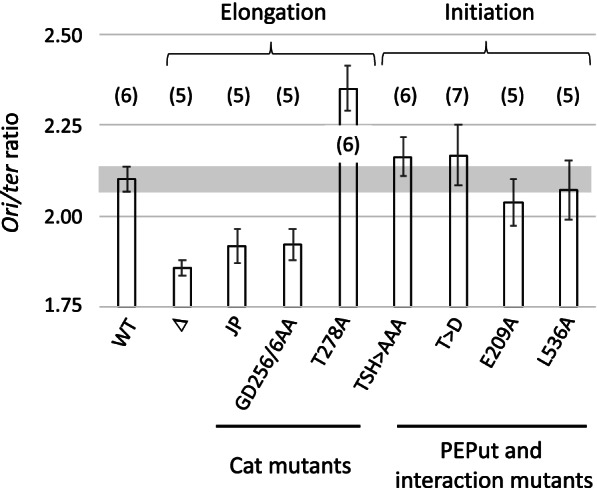
Primarily replication defect in pykA mutants. *Ori/ter* ratios were measured in cells replicating the chromosome from a plasmid replicon instead of the natural chromosomal replication origin. See Fig. 1 for details

Results showed a ratio typical to PykA+ *oriN*-dependent cells in PEPut and Cat-PEPut interaction mutants and a clearly reduced (*pykA*_*JP*_, *pykA*_*GD256/6AA*_) or increased (*pykA*_*T278A*_) ratio in Cat mutants (Fig. 4). Hence, PEPut and the Cat-PEPut interaction mutants are primarily affected in initiation while Cat mutants are primarily affected in elongation. This suggests that the commitment of PykA in replication involves two separate non-metabolic functions, one geared by PEPut and its interaction with Cat which affects initiation, and the other geared by Cat which affects elongation.

### PEPut is an inhibitor of initiation and Cat, a stimulator of elongation

**Fig. 5:**
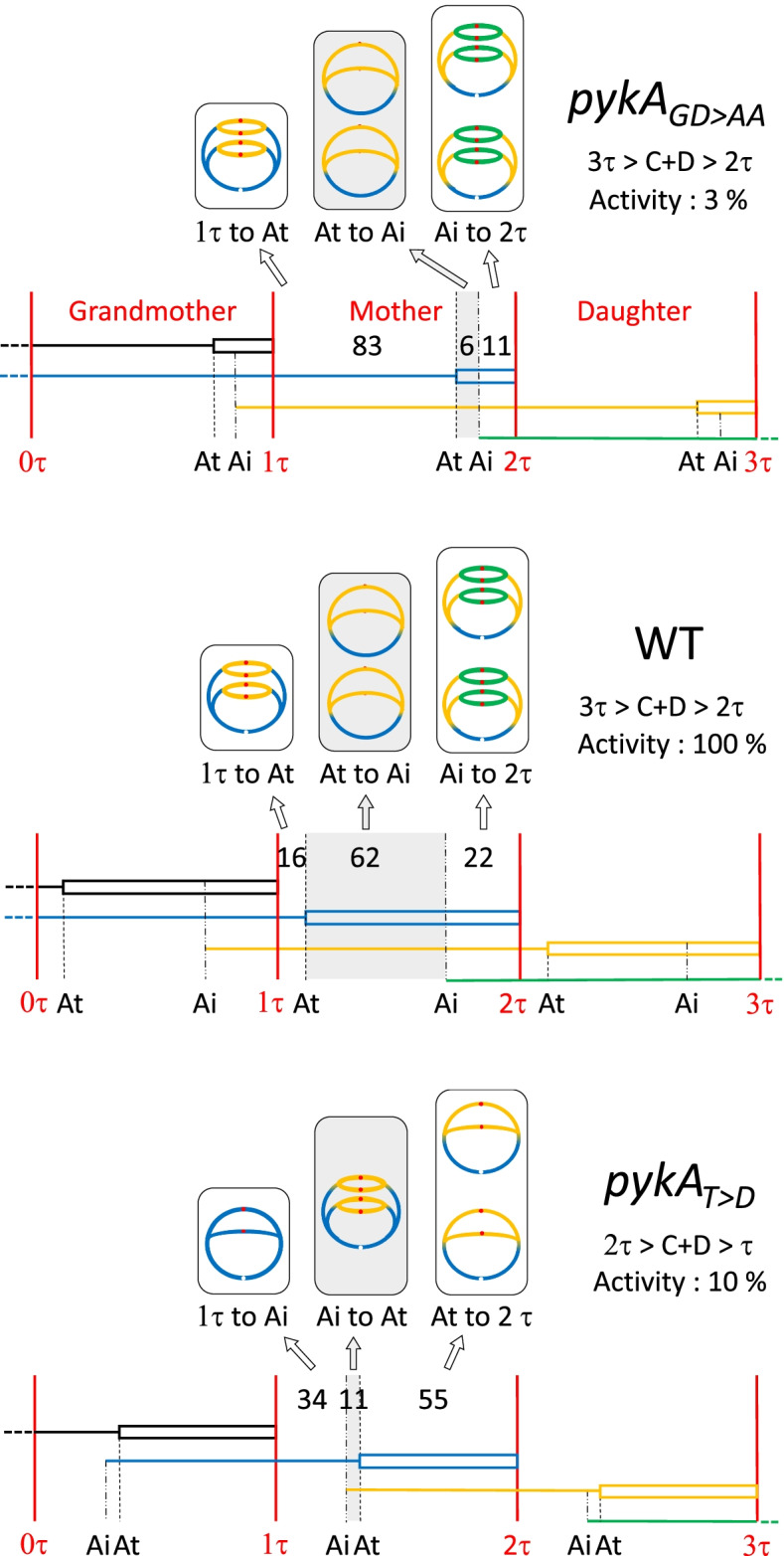
Cell cycle and replication pattern of wild-type, *pykA*_*GD245/6AA*_ and *pykA*_*T>D*_ cells. Vertical red lines stand for three successive generations: grandmother: 0τ to 1τ; mother: 1τ to 2τ; daughter: 2τ to 3τ; 28.4 min each). The C and D periods of cell cycles ending at time 1τ, 2τ, and 3τ are indicated as lines and boxes colored in black, blue and yellow, respectively. The cell cycle in green will end at time 4τ (not shown). Gray areas refer to replication periods spanning every cell cycle and numbers correspond to the proportion of cells in each period. Replication patterns typifying each period are given above the gray areas using the cell cycle color code. Ai: age of replication initiation; At: age of replication termination. Activity stand for pyruvate kinase activity

Microscopy studies showed that nucleoids are efficiently segregated in *pykA* mutants grown in MC (Fig. S5, top raw). Therefore, a cell cycle analysis was carried out in wild-type cells and *pykA* mutants using flow cytometry (runout experiments) and qPCR (marker frequency analysis), as described previously [97]. Representative results are given Fig. S5 (middle and bottom rows), and the complete set of data is summarized in Table S3. Results showed that PykA mutations can affect replication and cell cycle to remarkably high degrees and confirmed the prime importance of PEPut and Cat in initiation and elongation, respectively. More specifically and by comparison to the wild-type strain, cells lacking Cat (*pykA*_*ΔCat*_) and most (5/7) of the remaining Cat mutants replicate DNA remarkably slowly (600–740 bp/s instead of 830 bp/s in the wild-type strain) and have a marginal initiation defect (Table S3). In contrast, cells lacking PEPut (*pykA*_*ΔPEPut*_) and 3 other PEPut mutants (*pykA*_*T>D*_, *pykA*_*TSH>DDD*_, and *pykA*_*TSH>AAA*_) suffer from a dramatic initiation phenotype: they initiate replication in mother cells (i.e., in cells containing two origins) rather than in grandmother cells (i.e., in cells containing four origins) and their cell cycle lasts 2 instead of 3 generations (Table S3). Additionally, two of these mutants (*pykA*_*T>D*_ and *pykA*_*TSH>DDD*_) replicate DNA extremely fast (about 1000 bp/s). The dramatic effects the *pykA*_*T>D*_ (initiation) and *pykA*_*GD245/6AA*_ (elongation) mutations have on the cell cycle and replication pattern are highlighted Fig. 5.

Changes in nucleotide pools affect replication. In *pykA* mutants, such changes may result from an indirect effect of PykA mutations on CCM reactions producing nucleotide precursors or from a direct effect on ATP concentration (PykA produces ATP during the PEP to pyruvate conversion) and/or (d)NTPs production (the PykA catalytic site is promiscuous, using PEP as phosphoryl donor to phosphorylate (d)NDPs with various efficiencies [98]). If so, cells with similar defects in PykA activity would have similar pools and hence, similar replication phenotypes. Results do not support this assumption. Initiation is dramatically stimulated in four PEPut mutants having a low to moderate PykA activity (10–40%), while it is nearly unchanged in 13 mutants having a PykA activity ranging from 3 to 160% (Table S3). Moreover, very low, wild-type, and very fast fork speeds were found in strains with similar residual PykA activities (3–10%) (Table S3). Additionally, the metabolome analysis did not reveal significant changes in nucleotide pools (Fig. 3 and Table S2).

Collectively, our findings showed that PykA has two distinct replication functions with Cat acting as a stimulator of replication fork speed and PEPut, as an inhibitor of initiation. As the inhibitory activity of PEPut is massively abrogated in *pykA*_*TSH>AAA*_*, pykA*_*TSH>DDD*,_ and *pykA*_*T>D*_ cells, our results strengthen the notion that the TSH motif and its phosphorylation at *T* are of prime importance for initiation. Finally, given that replication phenotypes were found in cells fully proficient in “classical” replication control functions, this work showed that PykA is a new master regulator of replication.

### PEPut affects replication initiation and PykA catalytic activity in a medium-dependent manner

**Fig. 6:**
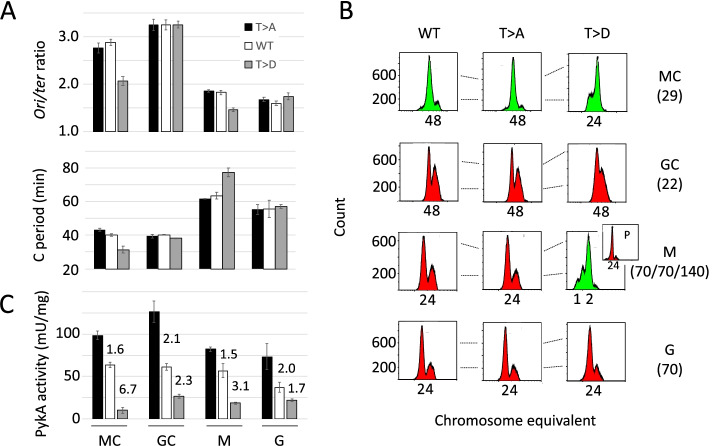
Replication and PykA catalytic activity analysis in wild-type, *pykA*_*T>A*_, and *pykA*_*T>D*_ cells grown in different media. **A** *Ori/ter* ratio and C period. **B** Runout DNA histograms. Numbers in brackets stand for generation times (min). **C** PykA catalytic activity. Numbers refer to ratios as follows: top row: *pykA*_*T>A*_/wild-type; bottom row: wild-type/*pykA*_*T>D*_. Data in A and C are from 3–6 biological repeats (mean and SD). Representative runout DNA histograms are shown in (**B**)

Results presented above showed that PykA is endowed with two replication functions in MC, a rich gluconeogenic medium. To investigate whether these functions are expressed in other nutritional conditions, replication parameters (*ori/ter* ratio, C period and number of *ori*/cell) of wild-type, *pykA*_*T>A*_, and *pykA*_*T>D*_ cells were assessed in another gluconeogenic broth (minimal medium supplemented with malate, M) and in two glycolytic media (minimal medium supplemented with glucose (G) or glucose + casa (GC)). Results showed wild-type parameters under the three tested conditions except in *pykA*_*T>D*_ cells grown in M (Fig. 6A, B). In this condition, a low *ori/ter* ratio, a low number of *ori*/cell, and an enlarged C period were found. However, while the three strains have similar growth rates in GC and G, the *pykA*_*T>D*_ strain grows poorly in M compared to wild-type and *pykA*_*T>A*_ cells (generation times 140 versus 70 min) (Fig. 6B and S5, left panel). This phenotype is a direct consequence of the *pykA*_*T>D*_ mutation, as catalytic mutants (*pykA*_*GD245/6AA*_ and *pykA*_*K220A*_) and *pykA* knockout cells grow as fast as the wild-type and *pykA*_*T>A*_ strains in M (Fig. S6, left panel). In order to further characterize the effect of the *pykA*_*T>D*_ mutation on replication in M, replication parameters of wild-type cells growing in proline at the similar rate as *pykA*_*T>D*_ in M were determined. Results (Fig. S6, right panel) showed that the mutant has a lower *ori/ter* ratio (1.3 versus 1.5), an enlarged C period (77 versus 58 min) and a lower number of *ori*/cell (compare runout DNA histograms) than wild-type cells in proline. In addition, they showed that the mutant likely suffers from an early initiation phenotype, as runout DNA histograms uncovered a significant proportion of non-initiated cells with 1 chromosome in *pykA*_*T>D*_ grown in M while the vast majority of non-initiated cells in proline cultures of the wild-type strain has 2 chromosomes. We concluded from these findings that PEPut inhibits initiation in gluconeogenic media (MC and M) through a mechanism abrogated by the *pykA*_*T>D*_ mutation. The lack of phenotype in *pykA*_*T>D*_ cells grown in GC and G either suggests that PykA does not impact initiation in glycolytic conditions or does so through a mechanism insensitive to the *pykA*_*T>D*_ mutation. Hence, the replication function of PEPut is medium dependent.

Next, we analyzed the role of PEPut in PykA catalytic activity in the strains and growth conditions used above. Results showed that PykA activity is increased in *pykA*_*T>A*_ and reduced in *pykA*_*T>D*_ contexts by comparison to wild-type cells in M, GC, and G (Fig. 6C) as it is in MC (Fig. 2) suggesting that T phosphorylation negatively regulates PykA catalytic activity in a large range of nutritional conditions. Interestingly, the level of T phosphorylation may be higher in glycolytic than in gluconeogenic media as the activity produced by wild-type cells is closer to that of *pykA*_*T>D*_ cells in glycolytic media and closer to that of *pykA*_*T>A*_ cells in gluconeogenic growth conditions (compare the ratios *pykA*_*T>A*_ versus wild-type and wild-type versus *pykA*_*T>D*_ Fig. 6C). It is inferred from this that the PEPut-driven regulation of PykA catalytic activity is sensitive to the polarity of the carbon flux travelling CCM.

Overall, our findings suggested that the level of T phosphorylation in the TSH motif varies with growth conditions and has two distinct functions: it negatively regulates PykA catalytic activity and alleviates the inhibitory activity of PEPut on replication initiation. However, while the former activity occurs at mid-levels of T phosphorylation, the latter activity may operate at high T phosphorylation levels.

### The purified PykA protein modulates replication activities of DnaE and DnaC

The effect of PykA on replication may involve the recruitment of the glycolytic enzyme to the initiation and/or elongation replication machineries. It was previously shown that PykA is produced at a rather high concentration [77] and is diffused throughout the cytoplasm [99]. Hence, for localizing PykA, we constructed strains encoding PykA-mCherry_BSU_ or mCherry_BSU_-PEPut fusions (wild-type or mutated) from an inducible promoter (*P*_*hyspank*_) at hundreds to thousands proteins per cell [46]. The constructions were inserted at an ectopic position (the *amyE* locus) in a context lacking or containing the natural *pykA* gene. Microscopy studies of living cells showed a diffused mCherry_BSU_ signal at all inducer concentrations (Fig. S7, top panels). However, upon cell fixation and at low inducer concentration (0-5 μM), irregular spotty signals were observed (Fig. S7, bottom panels). Unfortunately, we were unable to improve the fixative procedure and the quality of the images, preventing further analyses on PykA localization.

In order to investigate whether PykA affects replication by directly modulating the activity of replication enzymes, an in vitro approach was used. The PykA protein was heterologously expressed and purified, and control experiments showed that the purified PykA was metabolically active and formed the expected stable functional tetramer (Fig. S8). The effect of PykA was then tested on DnaC, DnaG, and DnaE, as these replication enzymes are genetically linked to PykA [42]. The DnaC helicase melts the duplex DNA at *oriC* during initiation and separates the DNA strands in replication forks during elongation. The DnaG primase synthesizes RNA primers at the origin and in replication forks. These primers are extended by DnaE to start leading-strand synthesis (which is mainly carried out by PolC) and to ensure partial or complete synthesis of the lagging strand. Previous studies showed that DnaC, DnaG, and DnaE form a ternary complex ensuring important roles during replication initiation and elongation [43, 45, 47, 49]. The three replication enzymes were purified and their activities were tested using previously described assays [43, 49] in the presence or absence of PykA. It is of interest to note here that these assays do not allow PykA activity (they lack the PykA substrates PEP and ADP) and probe key features of DNA elongation: chain polymerization and double-strand separation at forks. In contrast, they do not probe initiation as they give no information on origin melting and protein recruitment at melted origins.

**Fig. 7:**
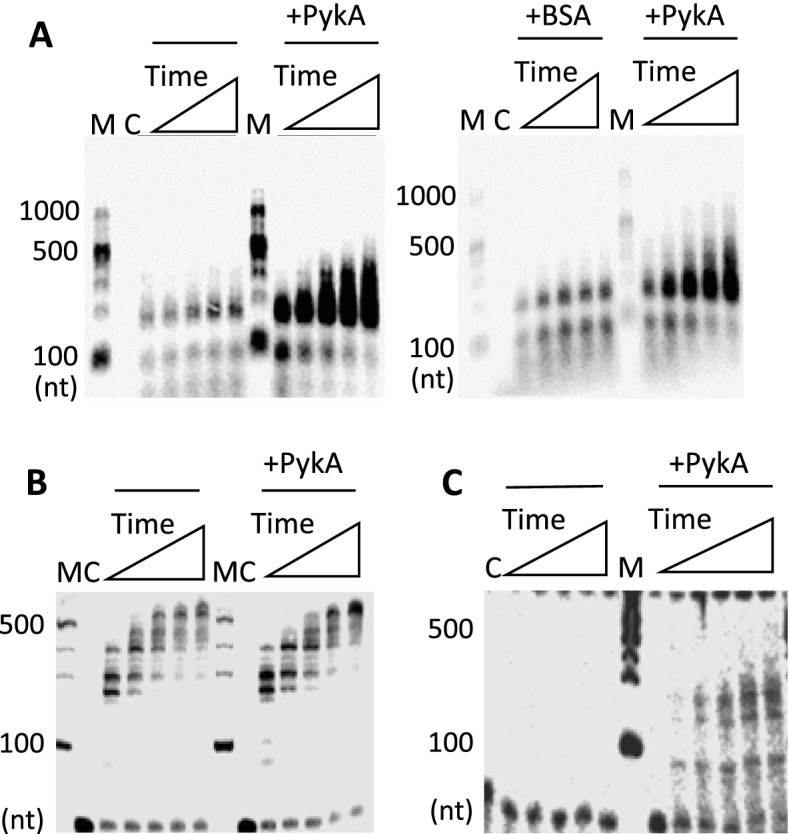
PykA stimulates the DNA polymerase activity of DnaE. **A** Representative alkaline agarose gels showing DnaE primer extension time courses (30, 60, 90, 120, and 150 s) with DnaE (10 nM) alone and in the presence or absence of PykA (40 nM tetramer) or BSA (40 nM), as described in “Methods”. Lanes M and C represent DNA size markers and the control radioactive substrate in the absence of any proteins, respectively. **B** Representative alkaline agarose gels (from three independent experiments) showing DnaE primer extension time courses (30, 60, 90, 120, and 150 s) with DnaE (10 nM) and in the presence or absence of PykA (40 nM tetramer), with molar ratios of DnaE monomer: DnaN dimer: HolB monomer:YqeN monomer:DnaX trimer:PykA tetramer set to 1:1:1:1:1:1, considering the oligomeric states of these proteins. **C** As in **B** with 2 nM DnaE and molar ratios of 1:1:1:1:1:1

Replication assays with DnaE were carried out at a polymerase concentration (10 nM) that produces a low amount of replication products in order to facilitate the detection of stimulatory effects. In reaction mixtures containing M13 ssDNA annealed to a 20-mer DNA oligonucleotide and DnaE in combination or not with equimolar concentrations of PykA, a substantial stimulation of DnaE polymerase activity by PykA was found in terms of both sizes and amounts of nascent DNA synthesized (Fig. 7A, left panel). A similar stimulation was observed with a 60-mer oligonucleotide annealed onto M13 ssDNA and a 15-mer oligonucleotide annealed onto a 110-mer oligonucleotide (Fig. S9A and data not shown). The stimulation was specific to PykA as it was not observed with equimolar amounts of BSA and M13 ssDNA primed with a 20-mer (Fig. 7A, right panel) or a 60-mer (Fig. S9B). The lack of DnaE stimulation by BSA was further confirmed at a 50-fold excess concentration over DnaE with the 20-mer primed M13 ssDNA (Fig. S9C) (note that the marginal stimulation observed at very high (500-fold) BSA excess is artifactual, acting likely as a blocking agent preventing adhesion of DnaE to the plastic reaction tubes). Titration experiments and gel shift assays showed that the stimulation of DNA synthesis by PykA was not due to a stimulation of DnaE binding to a primed template (Fig. S10).

Within the replisome, the polymerase activity of DnaE is stimulated by several proteins [43, 46, 49, 100], with DnaN being a potent stimulator. This ring-shaped protein is loaded at the 3’ end of primed sites by the clamp loader, encircles the DNA, and binds DnaE to form a stable complex that slides along the DNA template. In order to determine whether the polymerase activity of the DnaE-DnaN complex can be further stimulated by PykA, we carried out primer extension assays with DnaE, DnaN, proteins of the clamp loader (HolB, YqeN and DnaX), and PykA (Fig. 7B). As previously observed [43], we found that the polymerase activity of DnaE (10 nM) is strongly stimulated by DnaN and the clamp loader (compare the left panels of Fig. 7A, B). In this condition of high activity, the effect of PykA is unclear (Fig. 7B). However, this glycolytic enzyme may confer an additional stimulation to the DnaE activity as its presence leads at the last timepoints to a greater accumulation of large fragments and a clear reduction in labelled primer (Fig. 7B, compare with and without PykA). To confirm this, we carried out similar primer extension assays in the presence of lower, suboptimal concentrations of DnaE (2 nM), DnaN, clamp loader proteins, and PykA (in both sets of assay conditions, the molar ratios of proteins were kept identical). At this suboptimal DnaE concentration, no nascent DNA was detectable in the absence of PykA and significant amounts of nascent DNA fragments were synthesized by DnaE in the presence of PykA (Fig. 7C). This suggests that PykA stimulates the activity of DnaE even in conditions where its activity is strongly stimulated by DnaN. We concluded from these data that PykA stimulates the DnaE polymerase activity when the polymerase is slow (i.e., alone) and fast (i.e., in a complex with DnaN) probably via a direct interaction between PykA and DnaE. Since the purified PEPut domain does not affect DnaE activity (Fig. S11-12), the stimulation probably depends on a direct interaction between DnaE and the Cat domain or the interaction interface involves structural features of the PykA tetramer that are not preserved in the purified PEPut. The stimulation occurs with short (20-mer) and long (60-mer) primers suggesting that PykA may stimulate DnaE polymerase activity during extension of RNA primers generated by DnaG and during lagging-strand synthesis.

**Fig. 8:**
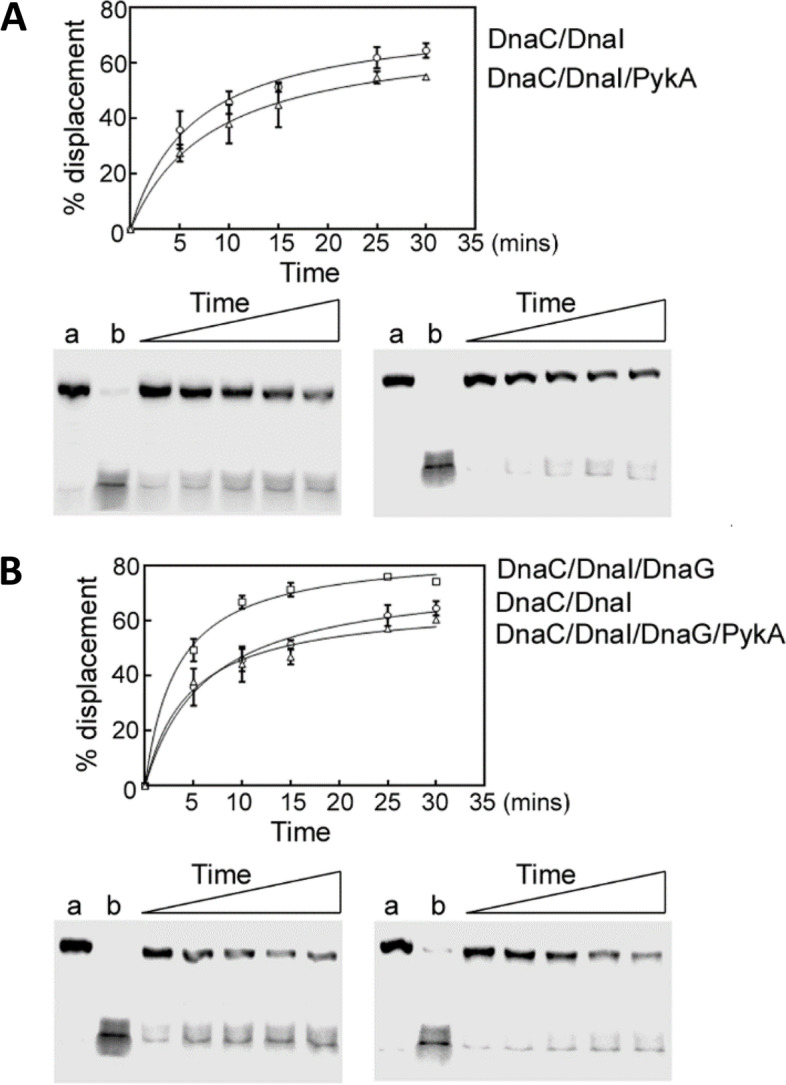
PykA directly inhibits the helicase activity of the helicase DnaC and via the primase DnaG. **A** Time courses (5, 10, 15, 25, and 30 min) showing the helicase activity of DnaC/DnaI in the presence or absence of PykA, as indicated. **B** Time courses (5, 10, 15, 25, and 30 min) showing the helicase activity of DnaC/DnaI in the presence or absence of PykA and/or DnaG, as indicated. The reactions were carried out as described in “Methods”. Representative native PAGE gels (from two independent experiments) are shown with lanes a and b representing control annealed and fully displaced (boiled) control DNA substrates. Data were plotted as a percentage of displaced primer versus time using GrapPad Prism 4 software. Error bars show the standard deviation from two independent repeat experiments

The helicase activity of DnaC was assayed by monitoring the displacement of a labelled 104-mer oligonucleotide annealed onto M13 ssDNA and forming a double-fork substrate with poly(dT) tails as previously [49]. To assemble a functional DnaC hexamer onto the DNA substrate, the helicase loader DnaI was added to reaction mixtures at equimolar concentrations. Results showed that DnaC is marginally inhibited in the presence of PykA (Fig. 8A). As we previously showed that DnaC activity is stimulated by DnaG [49], DnaG was added to the abovementioned reaction mixtures at equimolar concentrations. This analysis confirmed the stimulation of helicase activity by DnaG and showed that this stimulation is cancelled by PykA (Fig. 8B). Thus, in some contexts, PykA can significantly inhibit the helicase activity of DnaC. Collectively, these in vitro results suggest that PykA can modulate replication initiation and elongation through direct functional interactions with replication enzymes.

## Discussion

PykA is one of the most conserved and abundant proteins of the evolution tree and its ability to convert PEP and ADP into pyruvate and ATP during glycolysis is known in exquisite detail [73, 75]. Its activity is allosterically regulated through conformational changes within each subunit and between neighboring subunits promoted by the binding of effectors. Interestingly, an ever-increasing core of data showed that pyruvate kinases from bacteria to higher eukaryotes, akin other CCM enzymes, often ensure non-metabolic (moonlighting) functions in processes as diverse as transcription, viral replication, angiogenesis, pathogenicity, and tumorigenesis [60–62, 101, 102]. Here, we provide new insights on how the catalytic activity of *B. subtilis* PykA is regulated and demonstrate that this enzyme ensures non-metabolic functions important for replication initiation, elongation, and timing.

### Regulation of PykA catalytic activity

**Fig. 9:**
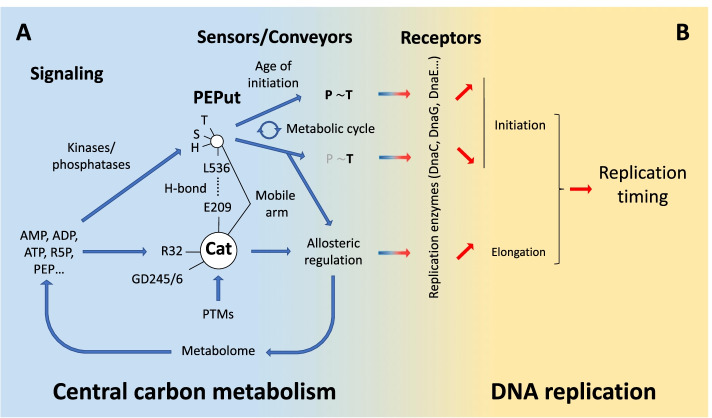
Intertwining model of PykA catalytic activity regulation and PykA-driven metabolic control of DNA replication. **A** Regulation of PykA catalytic activity. Several metabolites (PEP, ATP, ADP, AMP, R5P…) are used as signaling molecules for PykA regulation. They gear Cat-metabolite interactions, Cat-PEPut interaction, and TSH motif phosphorylation in PEPut. In response to these signals, PykA adopts different conformations with different types and levels of TSH motif phosphorylation. These multiple forms of PykA typify the cellular metabolic state and drive allosteric regulation of PykA catalytic activity. This regulation impacts the metabolome and the concentration of signaling metabolites. This mechanism can be implemented by posttranslational modifications (PTMs) of PykA. **B** Metabolic control of DNA replication by PykA. The ability of PykA to sense signaling metabolites (effectors and phosphoryl donors) and adopt multiple forms typifying the nutritional environment is used by cells to convey a metabolic signal to the replication machinery and regulate DNA synthesis in a large range of nutritional conditions. Top panel: metabolic control of initiation. (i) In response to the concentration of a phosphoryl donor and the activity of a kinase/phosphatase system, the phosphorylation level of the T residue in the TSH motif of PEPut varies. Initiation is inhibited at low- and activated at high phosphorylation levels. High phosphorylation level may be powered by a metabolic cycle causing periodic accumulation of the phosphoryl donor at the age of initiation. Bottom panel: metabolic control of elongation. The balance between the multiple forms of PykA is sensed by replication enzymes for modulating elongation. Previous data suggest that receptors of metabolic signals conveyed by PykA are the helicase DnaC, the primase DnaG, and the lagging strand polymerase DnaE which are essential for both replication initiation and elongation (see the “Background” section). The metabolic control of initiation and elongation help cells to properly time replication in the cell cycle in a large range of nutritional conditions

The *B. subtilis* PykA protein comprises the canonical catalytic domain (Cat) and an extra C-terminal peptide 110-amino-acid-long. This peptide, termed PEPut, is homologous to domains embedded in other metabolic enzymes and known to ensure catalytic and regulatory functions depending on phosphorylation of a conserved TSH motif (see  the "Background" section). Crystallographic studies suggested that Cat and PEPut interact through a hydrogen bond between E209 and L536 [74], and this interaction was proposed to assist heterotrophic effectors in stabilizing the active R-state conformation [75]. Here, we demonstrate that PEPut negatively regulates PykA catalytic activity up to 16-fold and that this process depends on the Cat-PEPut interaction, the TSH motif and its phosphorylation status (Fig. 2). A key determinant of this regulation may be the phosphorylation level of T in the TSH motif since the most dramatic changes in PykA activity occur in conditions mimicking the absence (T>A context, no inhibition) or massive (T>D context, strong inhibition) T phosphorylation (Fig. 2). Interestingly, T phosphorylation may vary with the polarity of the carbon flux travelling CCM, being higher in glycolytic than in gluconeogenic growth conditions (Fig. 6C). Our findings thus enforce the stabilization model of the active R-state conformation by Cat-PEPut interaction and further suggest that this stabilization is negatively regulated by T phosphorylation in the TSH motif. Hence, and assuming that knowledge in TSH motif phosphorylation prevails in *B. subtilis* PykA (see above), PykA regulation may depend on the concentration of phosphoryl donors (potentially PEP and ADP) and the activity of kinases/phosphatases for TSH phosphorylation, in addition to the concentration of PykA effectors (PEP, AMP, and ribose 5-phosphate). PykA can thus be viewed as a metabolic sensor mediating multiple interactions with allosteric regulators and exhibiting different TSH phosphorylation profiles depending on signaling metabolite concentration and CCM activity. This suggests that the thousands of PykA protein present in the *B. subtilis* cytoplasm exist in multiple forms and proportions that may vary with cellular metabolism for providing the active R-state PykA conformer at concentrations fulfilling biosynthetic needs and for conveying information on the nutritional environment as illustrated Fig. 9A. Additional posttranslational modifications (acetylation, succinylation…) prevalent in pyruvate kinases from bacteria to eukaryotes [91, 92] may further increase the diversity of PykA forms within cells enabling a higher level of PykA regulation and a more comprehensive information on the nutritional environment. This sophisticated regulation of PykA catalytic activity may provide an elegant solution to the problem posed by the constitutive and abundant production of PykA in a wide range of nutritional conditions [76, 77].

### PykA encodes initiation and elongation replication functions geared by PEPut and Cat, respectively

Replication phenotypes in *B. subtilis* cells lacking PykA were initially discovered in nutritional conditions where this glycolytic enzyme is important for growth and metabolism. Under these conditions, groups reported that *pykA* deletion allows (i) suppression of initiation and elongation defects due to thermosensitive mutations in the replication enzymes DnaC, DnaG, and DnaE (but not DnaI, DnaD, PolC, DnaX, and DnaN) [42, 43], (ii) stimulation of initiation [44], and (iii) alteration of the metabolic control of replication [15]. Here we show that *pykA* knockout cells cultivated in a medium (MC) where PykA is dispensable for metabolism and growth, also suffer from replication initiation and elongation defects (Fig. 1 and Table S3).

To gain insights into the PykA replication relationship, cell cycle parameters of Cat and PEPut mutants were monitored in MC. Results uncovered significant to strong replication phenotypes in most of the 18 tested mutants (Fig. 2 and Table S3). These phenotypes do not result from changes in PykA catalytic activity as this activity and replication parameters do not covary (Fig. 2). Additionally, they are not due to mere changes in cellular metabolism as the metabolome of *B. subtilis* in MC is resilient to changes in PykA catalytic activity (Fig. 3 and Table S2). In contrast, these phenotypes result from inactivation of either of two non-metabolic functions encoded by PykA: the one carried by PEPut which inhibits initiation, and the one carried by Cat which stimulates elongation. Interestingly, these moonlighting functions may operate in a medium-dependent manner (Fig. 6A,B). In vitro studies further showed that PykA functionally interacts with three enzymes essential for replication initiation and elongation. Indeed, the purified PykA protein stimulates the activity of the lagging-strand polymerase DnaE in conditions mimicking primer extension and lagging-strand synthesis, likely via physical contacts between Cat and DnaE (Fig. 7, S8-9 and S11). Moreover, the purified metabolic enzyme inhibits the helicase activity of DnaC and the stimulatory effect of DnaG on DnaC (Fig. 8).

Collectively, our report shows that PykA is endowed with a replication initiation and elongation function geared by PEPut and Cat, respectively, and that these functions may involve functional interactions with replication enzymes at *oriC* and replication forks. Although the strains used in this study are fully proficient in “classical” replication functions, most of the mutants lacking either PykA replication function exhibit remarkable cell cycle perturbations (Figs. 5 and 6 and Table S3). This surprising result suggests that the control geared by “classical” replication functions is far from sufficient to properly time DNA synthesis in the cell cycle and that an additional level of regulation by PykA is needed to meet this goal (at least in the gluconeogenic media used here). Therefore, we propose that PykA typifies a new family of replication control factor that operates in a medium-dependent manner along “classical” replication functions for gearing a metabolic control of DNA replication and for properly timing replication in the cell cycle.

### Basic principles of the metabolic control of replication by PykA

A targeted mutagenesis showed that the inhibitory activity of PEPut on initiation depends on the Cat-PEPut interaction, the TSH motif, and its phosphorylation status (Fig. 2 and Table S3). The importance of phosphorylation is highlighted by data suggesting that high T phosphorylation abrogates the initiation inhibitory activity of PEPut in gluconeogenic (but not glycolytic) growth conditions. In these conditions, *pykA*_*T>D*_ cells have an early initiation phenotype while initiation is properly regulated in the wild-type and *pykA*_*T>A*_ contexts (Fig. 6A, B). Assuming that key metabolites transiently accumulate through cell cycle progression in *B. subtilis* as in other systems [103–105], one can propose a model for initiation regulation by PEPut. In this model, initiation is inhibited for most of the cell cycle by a weakly T phosphorylated form of PEPut (equivalent to the wild-type and *pykA*_*T>A*_ contexts). However, late in the pre-initiation period, the initiation inhibitory activity of PEPut is alleviated by a massive and transient T phosphorylation (equivalent to *pykA*_*T>D*_ context). We propose that this transient phosphorylation occurs in response to the combined action of a cell cycle-dependent accumulation of a phosphoryl donor and of a strong kinase activity of a protein of the DUF299 family [85, 86] (a sequence analysis suggested that the poorly characterized protein YqfL is the *B. subtilis* DUF299 like protein. Its involvement in replication is under investigation). Intriguingly, the involvement of PEPut in initiation and PykA catalytic activity have similar requirements: they both depend on the Cat-PEPut interaction, the TSH motif, and its phosphorylation status. This suggests that the mechanism of the PEPut-driven metabolic control of initiation involves determinants of the mechanism of the PEPut-driven regulation of PykA catalytic activity, as illustrated Fig. 9. In this line of thinking, we propose that the system used for massive and transient T phosphorylation late in the pre-initiation period regulates PykA catalytic activity most of the cell cycle by ensuring a nutritional-dependent basal level of T phosphorylation.

The targeted mutagenesis also showed that residues R32, GD245/6, and amino acids 208–234 are of prime importance for Cat-driven stimulation of DNA elongation (Fig. 5 and Table S3). R32 and GD245/6 are involved in ATP/ADP and PEP binding [73] while the 208–234 region resides at the heart of the catalytic site and is genetically linked to the lagging-strand polymerase DnaE [42]. In vitro data further suggested that Cat interacts with the lagging-strand DnaE polymerase stimulating its activity (Fig. 7 and S11) and studies on different systems showed that lagging-strand synthesis gears fork speed [106–109]. Collectively, these results suggest that Cat stimulates elongation through a mechanism involving Cat-metabolites and Cat-DnaE interactions. As Cat-metabolite interactions induce complex conformational changes that cells use to allosterically regulate PykA catalytic activity in a wide range of nutritional conditions [73, 75], we propose that the mechanism of the Cat-driven metabolic control of elongation involves determinants of the mechanism of the Cat-driven regulation of PykA catalytic activity as illustrated Fig. 9. Again, these functions can be implemented by PykA posttranslational modifications [91, 92]. We conclude that cells use the ability of PykA to sense the metabolic status and adopt multiple forms typifying this status to convey metabolic information to the replication machinery and drive a metabolic control to DNA synthesis. We propose that the numerous CCM-replication links described in the literature (see  the "Background" section) highlight potential determinants of the metabolic control of replication in other systems.

The findings reported here show that side chain modifications disrupting PykA replication functions can dramatically disturb replication timing. As replication timing alterations cause accumulation of chromosomal lesions (double-strand breaks) with a high mutagenic potential [110], determinants of the metabolic control of replication may form a new family of genetic stability factors. Two hallmarks of cancer cells are aerobic glycolysis and a high rate of mutagenesis. Nowadays, cancer is thought to originate from mutations in oncogenes, tumor suppressors, and/or mutator genes, and other hallmarks, including their altered metabolism, are thought to be a result of these mutations rather than their cause [111, 112]. Extrapolating the results presented here from *B. subtilis* to humans, we speculate that CCM mutations (or changes in metabolite pools) that disrupt the metabolic control of replication may pave the way to genetic instability and may thus be an additional root cause of cancer initiation.

## Conclusions

Genome duplication is carried out once per cell cycle by a complex machinery tightly regulated by well-known, overlapping mechanisms. Failure in this regulation increases the risk of genetic instability and favors the emergence of diseases such as cancer. Since the 60s, genome duplication is also known to be under a metabolic control. The importance of this control for cell survival is unknown as well as its mechanism and interplay with classical replication control functions. Interestingly, an increasing core of data suggests that this mechanism may involve central carbon metabolism from bacteria to human. Accordingly, links between the enzyme ensuring the last reaction of glycolysis (the pyruvate kinase PykA) and replication in the bacterium *Bacillus subtilis* were found. Here, we show that the catalytic domain of *B. subtilis* PykA (Cat) stimulates DNA elongation while its C-terminal domain (PEPut) inhibits initiation and that these activities may involve functional interactions between Cat and enzymes involved in replication initiation and elongation. Very surprisingly, dramatic replication timing defects can occur in cells inactivated for either PykA replication activity and these activities depend on factors involved in regulation of PykA catalytic activity. We therefore propose that PykA typifies a novel family of replication regulator that acts in a nutrient-dependent manner to orchestrate the metabolic control of replication and replication timing. Since PykA’s replication functions and PykA regulation of catalytic activity share basic requirements, we propose that the mechanism allowing PykA to sense the cellular metabolic status and regulate its own activity is used by cells to control replication. We extrapolate from these results that the metabolic control of replication involves CCM enzymes endowed with a metabolic sensor activity from bacteria to human and that mutations that disrupt this control may disrupt replication timing and thus pave the way to genetic instability and diseases such as cancer.

## Methods

### Strains and plasmids

Strains and plasmids are listed in Supplementary Table S4. *E. coli* Stellar^TM^ competent cells (Clontech, Saint-Germain-en-Laye, France) were used as host for plasmid constructions. Most of the *B. subtilis* strains were constructed by transforming competent cells with genomic DNA (see Table S4 for details). Strains constructed using PCR products and plasmid DNA as transforming material are described below. The DGRM1173 strain containing the markerless *spsABCDEF* deletion was constructed as previously [95]. Genotypes of constructed plasmids and strains were checked by phenotypic analyses, endonuclease restriction, PCR analysis, and/or Sanger sequencing (Eurofins Genomics, Germany).

#### Construction of the pykA-tet strain (DGRM295)

In order to facilitate the construction of *pykA* mutants, we first inserted a *tet* gene immediately downstream from the transcription terminator of *pykA*. We first amplified with the Q5 high-fidelity DNA polymerase (New England Biolabs, Evry, Fr) the *tet* gene of plasmid pTB19 [113] and two chromosomal sequences flanking the site of insertion and containing the 3′ end of the *pykA* gene on the one hand and the *ytzA* plus the 3′ end of *ytvI* on the other hand. Then, we separated the PCR products from the parental genomic DNA by gel electrophoresis and purified the PCR fragments using the Monarch DNA Gel Extraction Kit (New England Biolabs, Evry, Fr). The purified fragments were then mixed and fused together using assembly PCR carried out with the Q5 high-fidelity DNA polymerase and the two primers flanking the fused fragment. This reaction depends on the addition of 20-bp sequences homologous to the 5′ and 3′ tails of the *tet* fragment to the internal side of the chromosomal PCR fragments. Competent cells of a wild-type strain cured of prophages (TF8A) were then transformed with the assembly PCR product and double crossover events integrating the *tet* gene downstream of *pykA* were selected on Tet containing plates (before plating, the *tet* gene was induced by incubating cells 1 h at 37 °C in the presence of 1.5 μg/mL Tet). A representative transformant, named DGRM295, was selected by PCR and DNA sequencing.

#### Construction of Cat and PEPut mutants

To generate Cat and PEPut mutants, pairs of PCR reactions were carried out using as template the genomic DNA of DGRM295 (*pykA-tet*). In each reaction, one external (i.e., in *pykA* or *ytvI*) primer and one mutagenic primer mapping in Cat or PEPut were used to generate PCR products with the desired *pykA* mutation at one end. PCR fragments were then assembled and the assembly products used to transformed TF8A competent cells, as described above (inactive pyruvate kinase mutants were selected on LB + Tet + Malate 0,4% w/v). Three representative transformants were selected by sequencing for nearly all constructions.

#### Construction of pykA mutants replicating the chromosome from oriN

To construct *pykA* mutants replicating the chromosome from *oriN*, competent cells of a TF8A derivative carrying *oriN* and the *cat* gene at the *spoIIIJ* locus and deleted for *oriC* (DGRM589, [44]) were transformed with genomic DNA of *pykA* mutants. Transformants were then selected on plates supplemented with appropriate antibiotic (and 0.4% w/v malate when the mutation inactivated PykA activity). Three representative transformants were generally selected. The presence of the *pykA* mutation was confirmed by sequencing while the presence of the *oriN-cat* structure and the *oriC* deletion were checked by measuring the size and sensitivity to *EcoRI* restriction of the corresponding PCR products.

#### Construction of strains encoding mCherry_BSU_ fusions

To generate strains encoding mCherry_BSU_ fusions, plasmids were first constructed (their sequence is available upon request). To construct pLJH225-Cm, a reverse PCR on pIC610 was carried out with the Q5 high-fidelity DNA polymerase to produce a linear fragment with the *Physpank* promoter (oriented outward) at one end and the PEPut ORF (oriented inward) at the other end. Using the same DNA polymerase and pBS1C3 as template, we then produced a PCR product containing the mCherry_BSU_ ORF fused to a linker (GGTGGAGGTGGATCT 3n) at the C-terminus. The fragment was engineered to carry at its extremities 20-bp-long sequences homologous to the ends of the pIC610 PCR product. The two fragments were then gel purified, ligated to each other using the NEBuilder Hifi DNA Assembly Master Mix (NEB, Evry, France) and the ligation product was used to transform Stellar^TM^ *E. coli* competent cells to AmpR. Transformants encoding the mCherry_BSU_-linker-PEPut fusion from *Physpank* were screened by colony PCR. The plasmid DNA was extracted from three representative positive clones and checked by DNA sequencing. A validated construct was named pLJH225-Cm and used to transform *B. subtilis* competent cells deleted for the natural *pykA* gene (DGRM25). A strain containing the fusion cassette inserted by a double crossover at the *amyE* locus was selected by PCR and named DGRM1110.

The construction of pLJH227 which contains the PykA-linker-mCherry_BSU_ fusion was carried as follows. First, we restricted the pDR111 DNA with *Nhe*I and *Hind*III and generated with the Q5 enzyme two PCR products, one containing the *pykA* ORF and its RBS region, the other the mCherry_BSU_ ORF fused in 5′ to the linker. The DNA fragments (containing appropriate 20 bp sequence homologies at their ends) were then gel purified and ligated using the NEBuilder Hifi DNA Assembly Master Mix. Upon transformation of Stellar^TM^ competent cells with the ligation mixture, selection on Amp containing plates, colony PCR screening, plasmid extraction, and DNA sequencing, we obtained pLJH227. To construct pLJH226, a similar strategy was followed using a PCR fragment containing the pykA_ΔPEPut_ ORF (and its RBS region) instead of the entire *pykA* ORF (and its RBS region). For generating pLJH226-Cm, the *Bgl*II fragment of pLJH226 containing the SpR marker and pUC sequences was replaced by the corresponding restriction fragment of pIC610 which contains a CmR marker in place of the SpR marker. The pLJH226-Cm and pLJH227 DNA were then used to transformed DGRM25-Km (*ΔpykA*) competent cells and representative transformants containing the fusion cassette inserted by a double crossover at the *amyE* locus were selected by PCR and named DGRM1109 (construction encoding the PykA-linker-mCherry_BSU_ fusion) or DGRM1111 (construction encoding the PykA_ΔPEPut_-linker-mCherry_BSU_ fusion).

### Growth conditions

Routinely, *B. subtilis* and *E. coli* cells were grown at 37 °C in LB with or without antibiotics at the following concentrations: spectinomycin (Sp, 60 μg/mL); tetracycline (Tet, 7.5 μg/ml); chloramphenicol (Cm, 5 μg/mL); phleomycin (Phl, 7.5 μg/mL); kanamycin (Kan, 5 μg/mL); ampicillin (Amp, 50 μg/mL). Malate (0.4% w/v) was added to LB for routine cultivation of *pykA* mutants. Other studies were carried out with cells grown at 37 °C in a minimal medium (K_2_HPO_4_: 80 mM; KH_2_PO_4_: 44 mM; (NH_4_)_2_SO_4_: 15 mM; C_6_H_5_Na_3_O_7_ 2H_2_0: 3, 4 mM; CaCl_2_: 50 mM; MgSO_4_: 2 mM; FeIII citrate: 11 μg/mL; MnCl_2_: 10 μM; FeSO_4_: 1 μM; FeCl_3_: 4 μg/mL; tryptophan: 0.01% w/v) supplemented with various carbon sources as follows (in w/v): MC medium: malate (0.4%) + enzymatic casein hydrolysate (casa, 0.02%); GC medium: glucose (0.4%) + enzymatic casein hydrolysate (casa, 0.02%); M medium: malate (0.4%); G medium: glucose (0.4%); C medium: casa (0.02%).

### Quantitative PCR

For monitoring *ori/ter* ratios, 6–14 cultures inoculated from generally three independent constructs were first grown overnight at 30 °C in MC supplemented with antibiotic. Upon saturation, cultures were diluted 1000-fold in the same medium without antibiotic and growth at 37 °C was carefully monitored using spectrophotometry. Samples for qPCR analysis were collected at low cell concentration (OD_600 nm_ = 0.06 to 0.15) to ensure that cell cycle parameters are determined in steady-state cells and are not affected by the approach to the stationary phase or by changes in medium composition. The genomic DNA was extracted as described previously [44] or using the PureLink Genomic DNA mini kit (Invitrogen by Thermo Fisher Scientific, Courtaboeuf, Fr). Every qPCR reaction was carried out using two technical repeats of 4 serial dilutions. A non-replicating control DNA (stage II sporlets, [114]) was analyzed simultaneously with the samples in about 1/4 of the qPCR plates. Reactions and amplifications were carried out as previously described in 1× SYBR qPCR Premix Ex Taq (Tli RNaseH Plus) (Ozyme, St Quentin en Yvelines, France) mix and on a Mastercycler®ep realplex (Eppendorf, Le Pecq, Fr) [44]. Ratios were normalized using the mean of about 125 measures of the non-replicating control DNA (0.5885 ± 0.006) and compared using the nonparametric Mann-Whitney *U* test (https://www.socscistatistics.com/tests/mannwhitney/default2.aspx) run at a significance level of *p* < 0.01 and a two-tailed hypothesis.

C periods were determined from three independent cultures and using 10 pairs of primers arranged regularly (from *oriC* to *terC*) along the right arm of the chromosome, as previously described [44]. Mean fork velocity was calculated using the C period (min) and the actual size of the TF8A genome (4,000,631 bp; this genome is deleted for prophages SPβ, PBSX, and *skin*).

### Flow cytometry analysis

Strains were grown as indicated in the previous section, and at OD_600nm_ = 0.1–0.15, chloramphenicol (200 μg/mL) was added to the cultures to impede replication initiation and cell division and allow completion of ongoing rounds of replication [115]. After 4 h of drug treatment, 1.5 mL of cells was fixed in filtered ethanol 70% v/v and stored at 4 °C. Stored cells were then washed twice in 1 mL of filtered Tris-buffered saline buffer (TBS 150) (20 mM Tris-HCl pH 7.5, 150 mM NaCl) and stained with Hoechst 33258 (1.5 μg/mL) for at least 30 min, as described elsewhere [97]. Flow cytometry analysis was carried out using a MoFlow Astrios cell sorter (Beckman Coulter, Life Sciences) equipped with a 355-nm krypton laser and a 448/59-nm bandpass filter used to collect Hoechst 33258 fluorescence data. Data were analyzed with the Kaluza software (Beckman Coulter, Life Sciences). We counted 100,000 events. Cell cycle parameters were determined as previously described [97].

### Pyruvate kinase activity measurement in crude extracts

Cells (25 mL; OD_600nm_ = 0.3) growing exponentially in MC were carefully collected by centrifugation (7 min; 4,922 RFC; room temperature) and resuspended in 75 μL of lysis buffer (Na_2_HPO_4_ 2H_2_O: 60 mM; NaH_2_PO_4_ 4H_2_O: 4 mM; KCl: 10 mM; MgSO_4_ 7H2O: 1 mM; DTT: 1mM; Lyzozyme: 0.1 mg/mL; DNase I: 40 U/mL). They were then incubated 20 min on ice, 5 min at 37 °C, and 15 min at room temperature. Crude extracts were then collected by centrifugation (10 min; 14,000 rpm; 4 °C). The PykA activity was determined using the colorimetric/fluorometric assay kit K709-100 (CliniScience, Nanterre, Fr) and fluorescence (Ex/Em = 535/587 nm) was assessed using a ClarioStar apparatus (BMG Labtech, Champigny-sur-Marne, Fr). Protein concentration was monitored using the standard Bradford assay.

### Microscopy

Cells were grown in MC medium as for qPCR analysis. For DNA segregation analysis, 1.5 mL of cells at OD_600nm_ = 0.15 were briefly (2 min) incubated in the presence of 1 μg/mL 4’,6’-diamidino-2-phenylindole (DAPI, Sigma-Aldrich, Saint-Quentin-Falavier, France) and/or 2 μg/mL FM4-94 (Invitrogen, Villebon-sur-Yvette, France) for nucleoid and membrane staining, respectively. They were then collected by gentle centrifugation at room temperature (11,000 rpm for 1 min), resuspended in 10 μL MC medium and immediately mounted on a glass slide covered with a 1.0% w/v agarose pad in MC. A 0.17-mm glass coverslip was then placed on top of the agarose pad. For mCherry_BSU_ fusion localization, cells prepared as above were collected by gentle centrifugation at room temperature (11,000 rpm for 1 min) and resuspended in MC or in a PBS solution containing 1% v/v paraformaldehyde (Alfa Aesar by Thermo Fisher Scientific, Kandel, Germany) (750 μL). Cells treated with paraformaldehyde were incubated 10 min at 4 °C. Samples treated or not with paraformaldehyde were then stained with DAPI, collected, and resuspended in 10 μL MC medium as above. Microscopy was carried out using an epifluorescence microscope (Zeiss, Axio Observer.Z1) with a × 100 magnification oil-immersion objective (Plan-APOCHROMAT Ph3) and a CMOS camera (Orca Flash 4.0 LT Hamamatsu). Digital images were acquired and analyzed using the Zen 2.6 (blue edition) software.

### Mass spectrometry

#### Metabolome preparation

Metabolite extraction was carried out from cells grown at 37 °C and an OD_600_ = 0.4 using a protocol adapted from the Metabolomics Service Protocols from the University of Glasgow (http://www.polyomics.gla.ac.uk/assets/downloads/MSMetabolomicsPrepCells-Aug2013.pdf) and modified as previously described [116]. For comparative analysis, *pykA*_*T>D*_ and wild-type cells were grown on solid medium and metabolomes were prepared as previously [94].

#### Chromatographic conditions

Analyses were conducted using a Dionex Ultimate 3000 Rapid Separation LC (Thermo Fisher Scientific). The method for metabolome analysis was previously reported [94]. For hydrogen/deuterium exchange (HDX) experiments, chromatographic conditions were also described [116].

#### HRMS analyses

High-resolution measurements were obtained with a VelosPro Orbitrap Elite mass spectrometer (Thermo Fisher Scientific) fitted with a heated electrospray ionization source operating in positive and negative ionization modes. The mass spectrometer settings were as previously reported [116]. Collision-induced dissociation spectra (CID) in resonant excitation conditions were acquired using data-dependent scanning function for identification purpose. Non-targeted CID experiments using data-dependent scan were conducted at 27% Normalized Collision Energy (NCE) in positive and negative modes. CID experiments to confirm the presence of Pse (Leg) in metabolome were conducted at 22% NCE in positive and negative modes.

#### Metabolomic data processing

LC/MS raw data were converted to mzXML using MSConvert 3.0.20338 [117] and processed with R package XCMS [118, 119] with an “in-house” modified KEGGREST (Tenenbaum and Maintainer, 2021) R function for automatic compound annotation. Multicore processing was made possible through R package BiocParallel (Morgan et al., 2021). Multivariate analyses were conducted using muma for data pretreatment (pareto scaling) and FactominR for PCA visualization [120, 121]. Metabolite identification was aided by MS^2^ interpretation and retention time matching to commercial reference standards. Metabolomics raw data are available in MetaboLights (https://www.ebi.ac.uk/metabolights/MTBLS4415) [122].

### Protein biochemistry

#### Replication enzymes

Replication enzymes DnaC, DnaG, DnaE, DnaN, HolB, YqeN, and DnaX were purified and tested as described previously [43, 49].

#### Helicase assays

Briefly, DnaC helicase assays were carried out with 633 nM (referring to monomer) each DnaC and DnaI, in 50 mM NaCl, 50 mM Tris-HCl pH 7.5, 10 mM MgCl_2_, 1 mM DTT, 2.5 mM ATP, and 2 nM DNA substrate, in the presence and absence of DnaG (313 nM) and/or PykA (633 nM monomer). The DNA substrate was constructed by annealing a 5′-^32^P radioactively labelled 100mer oligonucleotide (5′-CACACACACACACACACACACACACACACACACACACACACACACACACACACACACACCCCTTTAAAAAAAAAAAAAAAAGCCAAAAGCAGTGCCAAGCTTGCATGCC-3′) onto M13 ssDNA. The helicase activity was assayed by monitoring the displacement of the radioactive oligonucleotide from the M13 ssDNA through non-denaturing PAGE using 10% w/v polyacrylamide gels. Data were quantified using a Personal Molecular Imager with Quantity One 1-D analysis software (Bio-Rad) and analyzed with GraphPad Prism 4 software.

#### Polymerase assays

Time course polymerase assays were carried out by monitoring the DnaE primer extension activity using a 5′-^32^P radioactively labelled 20mer (5′-CAGTGCCAAGCTTGCATGCC-3′) or 60mer (5′-CAGTGCCAAGCTTGCATGCCTGCAGGTCGACTCTAGAGGATCCCCGGGTACCGAGCTCGA-3′) annealed to M13 ssDNA substrates (2 nM), in 50 mM Tris-HCl pH 7.5, 50 mM NaCl, 10 mM MgCl_2_, 1 mM DTT, 1 mM dNTPs, and 10 nM DnaE in the presence or absence of 40 nM PykA tetramer or 40 nM BSA. In some reactions with a lower, suboptimal 2 nM DnaE, 8 nM PykA tetramer was used. Nascent DNA was resolved through alkaline gel electrophoresis, as described before [43, 49]. Visualization and quantification were carried out using a Personal Molecular Imager with Quantity One 1-D analysis software (Bio-Rad) and data were analyzed with GraphPad Prism 4 software.

The effect of purified PEPut on the DnaE activity was investigated using a short DNA substrate comprising a 5′-^32^P radioactively 15mer oligonucleotide annealed onto a 110mer oligonucleotide as explained in Fig. S11.

### Cloning, expression, and purification of the B. subtilis PykA

#### Cloning of pykA

A DNA fragment of 1755 bp carrying the *B. subtilis pykA* gene was amplified from genomic DNA using the PyKAF (5′-TACTTCCAATCCAATGCAAGAAAAACTAAAATTGTTTGTACCATCG-3′) and PykAR (5′-TTATCCACTTCCAATGTTATTAAAGAACGCTCGCACG-3′) forward and reverse primers, respectively in colony PCR reactions using Q5 high-fidelity DNA polymerase. Typically, a *B. subtilis* single colony was suspended in 20 mL LB and grown at 37 °C until the optical density reached 0.4–0.8. Thereafter, colony PCR reactions were carried out with genomic DNA (10 μL) acting as the template at 10-fold dilution. The PCR reactions were carried out in a volume of 50 μL with 1 unit Q5 high-fidelity DNA polymerase, 0.5 μM PykAF and PykAR, and 0.25 mM dNTPs in 1XQ5 polymerase buffer. PCR products were cleaned up with the Clean-up kit (Thermo Scientific), resolved by agarose electrophoresis, gel extracted using the GeneJET Gel Extraction kit (Thermo Scientific) and cloned into the p2CT plasmid (gift by James Berger) using ligation independent cloning to construct the p2CT-BsuPykA expression vector. This vector codes for an N-terminally His-tagged/MBP PykA protein with the His-MBP tag removable by proteolysis using the TEV protease.

#### Expression of PykA

For heterologous expression of the *B. subtilis* PykA, the p2CT-BsuPykA expression vector was transformed into Rosetta(DE3) *E. coli*. Single colonies were used to inoculate two 600 mL 2xYT cultures, supplemented with 60 μL carbenicillin (50 mg/mL) in 2-Lt conical flasks. The flasks were incubated at 37 °C, with shaking (180 rpm) until the optical density reached 0.6–0.8. Expression of PykA was induced by the addition of 0.5 mM IPTG and a further 3 h of growth the cells were harvested by centrifugation at 3000*g* for 15 min. Cells were suspended in 30 mL of buffer A (500 mM NaCl, 50 mM Tris-HCl pH 7.5, 20 mM imidazole) supplemented with 1 mM PMSF and 50 μL protease inhibitor cocktail (Fischer), sonicated at 15 amplitude microns for 1 min, 4 times with 1 min intervals on ice in between. Then, benzonase (20 μL) was added to the cell lysate which was further clarified at 40,000*g* for 40 min. The soluble crude extract was clarified and filtered through a 0.22-μm filter.

#### Purification of PykA

The PykA protein was purified from the filtered crude extract using a combination of IMAC (Immobilized Metal Affinity Chromatography) and gel filtration. First, the filtered crude extract was loaded onto a 5-mL HisTrap HP column (GE Healthcare) equilibrated in buffer A. The column was washed thoroughly with buffer A and the PykA protein was eluted using gradient elution with buffer B (500 mM NaCl, 50 mM Tris-HCl pH 7.5, 1 M imidazole). The eluted PykA protein was collected and quantified spectrophotometrically (extinction coefficient 76,780 M^−1^ cm^−1^). TEV protease was added at 1:20 molar ratio while dialyzing the protein solution overnight in dialysis buffer (500 mM NaCl, 50 mM Tris-HCl pH 7.5) at 4 °C in order to remove the His-MBP tag. The untagged PykA protein was then loaded back onto a 5 mL HisTrap HP column equilibrated in buffer A and the flow-through containing the untagged PykA was collected. Finally, the PykA protein solution was spun concentrated to 5–7 mL using a vivaspin 10 kDa cut-off filter. EDTA was added to 1 mM and the PykA was then loaded onto a HiLoad 26/60 Superdex 200 Prep Grade gel filtration column (GE Healthcare) equilibrated in buffer C (500 mM NaCl, 50 mM Tris-HCl pH 7.5, and 1 mM EDTA). Fractions containing the PykA protein were pooled and the protein was quantified spectrophotometrically (extinction coefficient 8940 M^−1^ cm^−1^), aliquoted and stored in – 80 °C.

#### PykA activity assay

The activity of purified PykA was assayed coupling the PykA catalyzed reaction (conversion of phosphoenolpyruvate to pyruvate) to the conversion of pyruvate into lactate catalyzed by LDH (Lactate Dehydrogenase) in the presence of NADH at 25 °C. The oxidation of NADH to NAD was followed spectrophotometrically at 340 nm and this is directly proportional to the activity of PykA. The LDH-catalyzed reaction was first optimized to ensure that it does not become a limiting factor when measuring the activity of PykA. The PykA catalyzed reactions were carried out in 96-well plates using the reaction master mix (10 mM Tri-HCl pH 7.5, 10 mM MgCl_2_, 50 mM KCl, 0.5 mM NADH, 2 mM ADP, 9.375 × 10^−4^ mg/mL LDH, and 5.7 μg/mL PykA) at 25 °C, at increasing PEP concentrations (0, 0.001, 0.05, 0.1, 0.25, 0.5, 0.75, 1, 2, 4, 8, and 10 mM). Data were analyzed using GraphPad Prism 4 software to plot a Hill plot and a Michaelis-Menten plot from where *V*_max_, *K*_*m*_, and *n* (the Hill coefficient) were calculated using GraphPad Prism 4 software.

### Characterization of the oligomeric state of B. subtilis PykA

PykA assembles into a functional tetramer. The oligomeric state of the *B. subtilis* PykA was assessed by native mass spectrometry (MS) and gel filtration. Purified PykA protein was buffer exchanged into 200 mM ammonium acetate (pH 6.8) with a 75-μL Zeba (7k MWCO) desalting column. Protein (4 μM) was loaded onto a borosilicate emitter tip (prepared in-house) and back-fitted with a platinum wire electrode. Native mass spectra were acquired by nano-electrospray ionization on a modified Q-Tof instrument (Micromass) with 4.8 mbar backing pressure, capillary voltage of 1.3 kV, cone 30 V, source temperature 50 °C, collision 20 V, mass range 1500–15000 m/z, and a 32k quad profile for transmitting high m/z ions. The instrument was externally calibrated with NaCI (2 ng/μL). Mass spectra were processed with MassLynx v4.1 with minimal smoothing and background subtraction. Deconvoluted zero-charge spectra were generated using UniDec v4.2.2 with settings for low-resolution Q-ToF spectra.

The native MS spectrum revealed a very clean and extremely stable tetramer with miniscule amounts of dimer and monomer (Fig. S8E-F). Equally, comparative analytical gel filtration was consistent with a PykA tetramer as it eluted before the γ-globulin (158 kDa) and not as a monomer (62,314 Da) (Fig. S8G-H).

### Cloning, expression, and purification of the PEPut domain of B. subtilis PykA

#### Cloning of the PEPut domain

The DNA fragment coding for the PEPut domain, with the preceding 10 amino acids was isolated by PCR using genomic DNA and the pepF (5′-TACTTCCAATCCAATGCAGCACAAAATGCAAAAGAAGCT-3′) and pepR (5′-TTATCCACTTCCAATGTTATTAAAGAACGCTCGCACG-3′) primers and cloned into the p2CT plasmid, as described above for PykA*.* The resulting p2CT-PEPut expression construct produced an N-terminally His-MBP tagged PEPut protein. The His-MBP tag was removable by proteolysis using the TEV protease.

#### Expression and purification of PEPut

Expression and purification of the PEPut were carried out as described for the full length PykA protein but the last gel filtration step during purification was omitted. Quantification of the final untagged PEPut (MW 9582.8 Da) was carried out spectrophotometrically using the extinction coefficients 69,330 M^−1^ cm^−1^ (for the His-MBP tagged PEPut) and 1490 M^−1^ cm^−1^ (for the untagged PEPut after TEV protease treatment). Purified PEPut is shown in Fig. S11).

## Supplementary Information

**Additional file 1: Fig. S1**. Key amino-acids of the Cat and PEPut domains of PykA. **A.** Cat domain analysis. Clustalw and Chimera analysis of the pyruvate kinase of *B. subtilis* (PykA), human cells (PKM2) and *Mycobacterium tuberculosis* (PYK) identified key amino acids of the catalytic site of the *B. subtilis* protein. **B.** PEPut domain analysis. Alignment of the PEPut domain of PykA to related domains of various metabolic enzymes. The red arrow highlights the conserved LTSH motif (coordinates 536-539). **Fig. S2**. Effect of Cat and PEPut mutations on growth in MC. Wild-type and *pykA* mutants were first grown over-night in MC supplemented with antibiotic when appropriate. Upon saturation, cultures were diluted 1000-fold in the same medium without antibiotic and growth was monitored spectrophometrically. Left panel: Analysis of catalytic mutants (*pykA*_*Δcat*_, *pykA*_*R32A*_, *pykA*_*R73A*_, *pykA*_*K220A*_, *pykA*_*GD245/6AA*_, *pykA*_*T278A*_, *pykA*_*JP*_). Right panel: Analysis of PEPut and Cat-PEPut interaction mutants (*pykA*_*ΔPEP*_, *pykA*_*T>A*_, *pykA*_*S>A*_, *pykA*_*H>A*_, *pykA*_*TSH>AAA*_, *pykA*_*T>D*_, *pykA*_*S>D*_, *pykA*_*H>D*_, *pykA*_*TSH>DDD*_, *pykA*_*E209A*_, *pykA*_*L536A*_). Controls: TF8A (wild-type) and *ΔpykA*. **Fig. S3.** Analysis of NTP in the metabolome of wild-type and *pykA*_*T>D*_ cells. ATP, GTP and CTP were detected in the positive ionization mode. UTP was detected in the negative ionization mode. Note that TTP signals were too low for quantifications. Data correspond to 3 independent extractions (solid cultures).*, *p* > 0.05 ; **, *p* < 0.05 (Welch's T-test). Values in bold indicate the fold change for each metabolite (WT vs *pykA*_*T>D*)._ **Fig. S4.** LC/MS analysis of legionaminic acid in the metabolome. **A.** Extracted ion chromatogram (EIC) corresponds to the deprotonated molecule [M-H]^-^ at *m/z* 333.1303 (5 ppm accuracy). **B.** Zoom on the mass spectrum of legionaminic acid in the negative mode. **C.** Collision Induced dissociation (CID) spectrum of legionaminic acid in the negative mode at 22% Normalized Collision Energy (NCE). **D.** CID spectrum of legionaminic acid in the positive mode at 22% NCE. **E and F.** Zoom on the mass spectrum of the deuterated forms of legionaminic acid in the negative and positive ionization mode, respectively. **G and H.** Comparison of legionaminic acid (G) and CMP-legionaminic (H) acid contents in wild-type (WT), *ΔspsE* and *ΔspsF* cells, respectively. Data correspond to 3 independent extractions (liquid cultures). ***, p < 0.001; **, p < 0.01 (Welch's T-test). **Fig. S5.** Representative cell cycle results in Cat and PEPut mutants. Top raw: Microscopy of exponentially growing cells stained with FM4-64 (membrane staining, red) and DAPI (nucleoid staining, blue). Middle raw: Representative runout DNA histograms (experiments were reiterated 3-12 times). Bottom raw: Representative marker frequency analysis along the right arm of the chromosome (experiments were reiterated at least three times). **Fig. S6.** Cell cycle parameters of wild-type and *pykA*_*T>D*_ cells grown in proline and malate, respectively. Left panel: Growth in malate of wild-type (WT), *ΔpykA* (*Δ*), *pykA*_*T>D*_ (T>D) and other pykA mutants (*pykA*_*T>A*_, *pykAGD*_*245/6AA*_ and *pykA*_*K220A*_, blue lines). Right panel: Runout DNA histograms and cell cycle parameters. **Fig. S7**. PykA-mCherry_BSU_ localization. Strains deleted for the natural *pykA* gene and encoding the PykA-mCherry_BSU_ fusion from an inducible promoter (*P*_*hyspank*_) were grown in MC and microscopy analysis was carried out at OD_600nm_ = 0.1 to 0.2. Top raw: analysis of the mCherry_BSU_ signal produced at different IPTG concentrations. Bottom raw: analysis of cells grown in the absence of IPTG, fixed in a 1x PBS solution supplemented with 1% paraformaldehyde and stained with DAPI. Scale bar: 4 μm. Similar results were obtained with fusions mutated in the Cat or PEPut domain of PykA. **Fig. S8.** PykA purification and characterization of its function and oligomeric state. **A.** SDS-PAGE (15% polyacrylamide gel) showing over-expression of the 6His-MBP-PykA in Rosetta (DE3) *E. coli.* The soluble expressed tagged PykA protein is shown in a red rectangular in lane 4, whereas lanes M, 1, 2 and 3 show molecular weight standards, the control uninduced insoluble fraction, the control uninduced soluble fraction and the IPTG-induced insoluble fraction, respectively. **B.1.** SDS-PAGE (15% polyacrylamide gel) showing fractions from the first IMAC purification step of PykA. From left to right, lanes represent molecular weight standards (M), the flow-through (1), the eluted tagged PykA (2), the overnight TEV treated tagged PykA (3), the flow-through fractions containing untagged PykA from the second IMAC step after TEV proteolysis (4-10). **B.2.** SDS-PAGE (15% polyacrylamide gel) showing the final gel filtration column (HiLoad 26/60 Superdex 200 Prep Grade Gel Filtration Column). From left to right, lanes represent molecular weight standards (M) and fractions of the size exclusion chromatography (1-9). **C.** The graph shows a Hill plot for the activity of PykA at 25°C. The Rate/(Vmax-Rate) (Y-axis) was plotted against the PEP substrate concentration (X-axis) using GraphPad Prism 4 software and the Vmax (19.3 μmol/min), Km (2.7 mM) and the Hill coefficient n (0.8111) values are shown below the graph. The n value is <1 indicating negative cooperative binding of PykA to its PEP substrate. **D.** The graph shows a Michaelis-Menten plot for the activity of PykA at 25°C. The initial rate of the reaction (Y-axis) was plotted against the PEP substrate concentration (X-axis) using GraphPad Prism 4 software and the Vmax (16.3 μmol/min) and Km (1.7 mM) values are shown below the graph. **E.** A native mass spectrum showing the PykA tetramer and miniscule amounts of the dimer and monomer. The theoretical mass of the PykA monomer (62,314.9 Da), dimer (124,629.8 Da) and tetramer (249,259.6 Da). Native mass spectrometry showed that PykA was found to be predominantly tetrameric (250,092 ± 72 Da), with very low abundance dimer (124,811 ± 38 Da) and monomer (62,437 ± 8 Da) peaks. **F.** Collision induced dissociation of the 33+ charge state of the tetramer shows it to be very stable in the gas-phase, with no apparent dissociation to lower-order oligomers. **G.** Comparative analytical gel filtration of the PykA tetramer against molecular weight standards (Thyroglobulin 670 kDa, g-globulin 158 kDa, ovalbumin 44 kDa, myoglobin 17 kDa and vitamin B12 1.3 kDa) through a Superdex 200 10/300 GL prepacked Tricorn gel filtration column (GE Healthcare). **H.** Selectivity trendline constructed from the molecular weight standards (shown in graph G) for the estimation of the PykA MW. The x axis is in logarithmic scale. Graphpad was used for plotting the data points. The theoretical value of our PykA (249,259.6 Da) is close to the estimated (285,000 Da) which along with the MS data verifies the tetramer in solution. K_d_ is the equilibrium distribution coefficient. The numbers (1-5) on the data points correspond to the proteins shown in graph G. **Fig. S9**. Stimulation of DnaE activity by PykA but not by BSA. **A.** Primer extension assays monitoring the extension of a 5′-^32^P-radioactively labelled 60mer DNA primer annealed onto M13 ssDNA over time by the *B. subtilis* DnaE. The activity of DnaE polymerase (10 nM) was monitored in the presence and absence of PykA (10 nM, tetramer) through a time course (30-150 sec). Lanes in the gels from left to right indicate: (M): DNA-ladder and then the time course (0, 30, 60, 90, 120 and 150 sec) depicted by the rectangular triangle. **B.** Primer extension assays as above with or without 10 nM (monomer) BSA instead of PykA. **C.** DnaE (1 nM) polymerase activity at increasing BSA concentrations (0, 5, 50, 500 nM), as indicated by the rectangular triangle, monitored by alkaline agarose electrophoresis. The DNA substrate is a labelled 20mer (5′-CAGTGCCAAGCTTGCATGCC-3′) primer annealed onto ssM13 ssDNA (2nM). The primer extension reaction was carried out for a longer time than above (5 min instead of 30-150 sec) and the film was over-exposed to compensate for the lower DnaE concentration. The assay was carried out at 37 °C in 50 mM Tris-HCl 7.5, 50 mM NaCl, 10 mM MgCl_2_ mM DTT, 1 mM dNTPs. No stimulation of the DnaE polymerase activity was observed in the presence of 5 and 50 nM BSA. The marginal stimulation observed at 500 nM BSA excess is likely because at this high concentration, BSA acts as a blocking agent preventing adhesion of DnaE to the plastic reaction tubes. **Fig. S10.** Stimulation of DnaE activity by PykA does not result from stimulation of DnaE binding to primed templates. EMSA investigation of the effect of PykA on the DNA binding of DnaE polymerase. The DNA substrate was constructed by annealing a 5′-^32^P-radioactively labelled 15mer (5′-AAGGGGGTGTGTGTG-3′) primer annealed onto a 30mer (5′-ACACACACACACACACACACACACCCCCTT-3′) oligonucleotide. Binding reactions were carried out with 1 nM DNA substrate, DnaE (500nM) and increasing concentrations (0, 12.5, 125 and 1,250 nM tetramer) of PykA, as indicated by the rectangular triangle for 10 min at 37°C in 50 mM NaCl, 10 mM MgCl_2_, 50 mM Tris-HCl pH 7.5. Lanes C and PykA represent the radioactive substrate in the absence of any proteins and in the presence of PykA (1,250 nM tetramer), respectively, showing that PykA does not bind to the DNA substrate. No stimulation of DnaE binding to DNA was observed in the presence of increasing concentrations of PykA indicating that PykA does not enhance the DNA binding activity of DnaE. **Fig. S11.** PEPut purification. **A.** SDS-PAGE showing overexpression of the His-MBP tagged PEPut in Rosetta (DE3) *E. coli*. From left to right, lanes show protein MW markers (M), the insoluble uninduced (1), soluble uninduced (2), insoluble induced (3) and soluble induced (4) fractions. The expressed soluble His-MBP tagged PEPut is shown by a red rectangular. **B.** SDS-PAGE showing the final purified untagged PEPut after removal of the His-MBP tag with TEV proteolysis. Lanes from left to right show protein MW markers (M) and fractions from the flow through the HisTrap column containing the pure untagged PEPut (lanes 1,2 and 3). **Fig. S12.** PEPut does not stimulate DnaE activity. Primer extension time course (30, 60, 90, 120 and 150 sec) assays using a primed DNA substrate (133 pM) constructed by annealing a radioactively labelled 5′-^32^P 15mer primer (5′-AAGGGGGTGTGTGTG-3′) onto a 110mer oligonucleotide (5′-CACACACACACACACACACACACACACACACACACACACACACACACACACACACACACCCCTTTAAAAAAAAAAAAAAAAGCCAAAAGCAGTGCCAAGCTTGCATGCC-3′), at suboptimal 25 pM DnaE concentration (left), in the presence of 25 pM PykA tetramer (middle) and 25 pM PEPut domain monomer (right). At this suboptimal DnaE concentration, there is no detectable DnaE primer extension activity in the absence of PykA but clear activity is visible in the presence of PykA. By comparison, no DnaE activity is detectable in the presence of the purified PEPut domain. These data show that full length PykA stimulates the DnaE activity while the PEPut domain alone does not.

**Additional file 2: Tables S1-S4**.

## Data Availability

All data generated and analyzed in this study are available, either in this published article or the supplementary material or in the MetaboLights repository (https://www.ebi.ac.uk/metabolights/MTBLS4415) [122]. Strains and plasmids are available upon request from the authors as well as sequence details.

## References

[1] Siddiqui K, On KF, Diffley JFX (2013). Regulating DNA replication in eukarya. Cold Spring Harb Perspect Biol. Cold Spring Harbor Lab.

[2] Jameson K, Wilkinson AJ (2017). Control of initiation of DNA replication in *Bacillus subtilis* and *Escherichia coli*. Genes..

[3] Bipatnath M, Dennis PP, Bremer H (1998). Initiation and velocity of chromosome replication in *Escherichia coli* B/r and K-12. J Bacteriol.

[4] Helmstetter CE, Neidhart FC, Curtis RI, Ingraham EC, Lin KB (1996). *Timing of synthetic activities in the cell cycle*. *Escherichia coli* and *Salmonella*.

[5] Schaechter M, Maaloe O, O KN. (1958). Dependency on medium and temperature of cell size and chemical composition during balanced growth of *Salmonella typhimurium*. J Gen Microbiol.

[6] Sharpe ME, Hauser PM, Sharpe RG, Errington J (1998). *Bacillus subtilis* cell cycle as studied by fluorescence microscopy: constancy of cell length at initiation of DNA replication and evidence for active nucleoid partitioning. J Bacteriol.

[7] Buchakjian MR, Kornbluth S (2010). The engine driving the ship: metabolic steering of cell proliferation and death. Nat Rev Microbiol.

[8] Burnetti AJ, Aydin M, Buchler NE (2015). Cell cycle Start is coupled to entry into the yeast metabolic cycle across diverse strains and growth rates. Mol Biol Cell.

[9] Ewald JC (2018). How yeast coordinates metabolism, growth and division. Curr Opin Microbiol.

[10] Klevecz RR, Bolen J, Forrest G, Murray DB (2004). A genomewide oscillation in transcription gates DNA replication and cell cycle. Proc Natl Aca Sci USA.

[11] Papagiannakis A, Niebel B, Wit EC, Heinemann M (2017). Autonomous metabolic oscillations robustly gate the early and late cell cycle. Mol Cell.

[12] Tu BP, Kudlicki A, Rowicka M, McKnight SL (2005). Logic of the yeast metabolic cycle: temporal compartmentalization of cellular processes. Science..

[13] Yu F-X, Dai R-P, Goh S-R, Zheng L, Luo Y (2009). Logic of a mammalian metabolic cycle: an oscillated NAD+/NADH redox signaling regulates coordinated histone expression and S-phase progression. Cell Cycle.

[14] Flåtten I, Fossum-Raunehaug S, Taipale R, Martinsen S, Skarstad K (2015). The DnaA protein is not the limiting factor for initiation of replication in *Escherichia coli*. PLoS Genet.

[15] Murray H, Koh A (2014). Multiple regulatory systems coordinate DNA replication with cell growth in *Bacillus subtilis*. PLoS Genet.

[16] Mathews CK (2015). Deoxyribonucleotide metabolism, mutagenesis and cancer. Nat Rev Cancer.

[17] Maya-Mendoza A, Moudry P, Merchut-Maya JM, Lee M, Strauss R, Bartek J (2018). High speed of fork progression induces DNA replication stress and genomic instability. Nature..

[18] Hu C-M, Tien S-C, Hsieh P-K, Jeng Y-M, Chang M-C, Chang Y-T (2019). High glucose triggers nucleotide imbalance through O-GlcNAcylation of key enzymes and induces KRAS mutation in pancreatic cells. Cell Metab.

[19] Lu M, Campbell JL, Boye E, Kleckner N (1994). SeqA: a negative modulator of replication nitiation in *E. coli*. Cell..

[20] Ishida T, Akimitsu N, Kashioka T, Hatano M, Kubota T, Ogata Y (2004). DiaA, a novel DnaA-binding protein, ensures the timely initiation of *Escherichia coli* chromosome replication. J Biol Chem.

[21] Baranska S, Glinkowska M, Herman-Antosiewicz A, Maciag-Dorszynska M, Nowicki D, Szalewska-Palasz A (2013). Replicating DNA by cell factories: roles of central carbon metabolism and transcription in the control of DNA replication in microbes, and implications for understanding this process in human cells. Microb Cell Fact.

[22] Boye E, Nordström K (2003). Coupling the cell cycle to cell growth. EMBO Rep.

[23] Wang JD, Levin PA (2009). Metabolism, cell growth and the bacterial cell cycle. Nat Rev Microbiol.

[24] Du Y-CN, Stillman B (2002). Yph1p, an ORC-interacting protein: potential links between cell proliferation control, DNA replication, and ribosome biogenesis. Cell.

[25] Beaufay F, Coppine J, Hallez R (2021). When the metabolism meets the cell cycle in bacteria. Curr Opin Microbiol.

[26] Fernández-Coll L, Maciag-Dorszynska M, Tailor K, Vadia S, Levin PA, Szalewska-Palasz A (2020). The absence of (p)ppGpp renders initiation of *Escherichia coli* chromosomal DNA synthesis independent of growth rates. mBio..

[27] DeNapoli J, Tehranchi AK, Wang JD (2013). Dose-dependent reduction of replication elongation rate by (p)ppGpp in *Escherichia coli* and *Bacillus subtilis*. Mol Microbiol.

[28] Hernandez JV, Bremer H (1993). Characterization of RNA and DNA synthesis in *Escherichia coli* strains devoid of ppGpp. J Biol Chem.

[29] Chubukov V, Gerosa L, Kochanowski K, Sauer U (2014). Coordination of microbial metabolism. Nat Rev Microbiol.

[30] Liu GY, Sabatini DM (2021). mTOR at the nexus of nutrition, growth, ageing and disease. Nat Rev Mol Cell Biol.

[31] Hughes P, Landoulsi A, Kohiyama M (1988). A novel role for cAMP in the control of the activity of the *E. coli* chromosome replication initiator protein, DnaA. Cell..

[32] Maciąg M, Nowicki D, Jannière L, Szalewska-Pałasz A, Wegrzyn G (2011). Genetic response to metabolic fluctuations: correlation between central carbon metabolism and DNA replication in *Escherichia coli*. Microb Cell Fact.

[33] Maciag-Dorszynska M, Ignatowska M, Jannière L, Wegrzyn G, Szalewska-Pałasz A (2012). Mutations in central carbon metabolism genes suppress defects in nucleoid position and cell division of replication mutants in *Escherichia coli*. Gene..

[34] Tymecka-Mulik J, Boss L, Maciag-Dorszynska M, Matias Rodrigues JF, Gaffke L, Wosinski A (2017). Suppression of the *Escherichia coli dnaA46* mutation by changes in the activities of the pyruvate-acetate node links DNA replication regulation to central carbon metabolism. PLoS One.

[35] Zhang Q, Zhou A, Li S, Ni J, Tao J, Lu J (2016). Reversible lysine acetylation is involved in DNA replication initiation by regulating activities of initiator DnaA in *Escherichia coli*. Sci Rep.

[36] Krause K, Maciag-Dorszynska M, Wosinski A, Gaffke L, Morcinek-Orłowska J, Rintz E (2020). The role of metabolites in the link between DNA replication and central carbon metabolism in *Escherichia coli*. Genes..

[37] Bergé M, Pezzatti J, Gonzalez-Ruiz V, Degeorges L, Mottet-Osman G, Rudaz S (2020). Bacterial cell cycle control by citrate synthase independent of enzymatic activity. eLife..

[38] Laffan J, Firshein W (1987). Membrane protein binding to the origin region of *Bacillus subtilis*. J Bacteriol.

[39] Laffan J, Firshein W (1988). Origin-specific DNA-binding membrane-associated protein may be involved in repression of initiation in *Bacillus subtilis*. Proc Natl Aca Sci USA.

[40] Stein A, Firshein W (2000). Probable identification of a membrane-associated repressor of *Bacillus subtilis* DNA replication as the E2 subunit of the pyruvate dehydrogenase complex. J Bacteriol.

[41] Noirot-Gros M-F, Dervyn E, Wu LJ, Mervelet P, Errington J, Ehrlich SD (2002). An expanded view of bacterial DNA replication. Proc Natl Aca Sci USA.

[42] Jannière L, Canceill D, Suski C, Kanga S, Dalmais B, Lestini R (2007). Genetic evidence for a link between glycolysis and DNA replication. PLoS One.

[43] Paschalis V, Le Chatelier E, Green M, Képès F, Soultanas P, Jannière L (2017). Interactions of the *Bacillus subtilis* DnaE polymerase with replisomal proteins modulate its activity and fidelity. Open Biol.

[44] Nouri H, Monnier A-F, Fossum-Raunehaug S, Maciag-Dorszynska M, Cabin-Flaman A, Képès F (2018). Multiple links connect central carbon metabolism to DNA replication initiation and elongation in *Bacillus subtilis*. DNA Res.

[45] Dervyn E, Suski C, Daniel R, Bruand C, Chapuis J, Errington J (2001). Two essential DNA polymerases at the bacterial replication fork. Science..

[46] Le Chatelier E, Becherel OJ, d'Alencon E, Canceill D, Ehrlich SD, Fuchs RPP (2004). Involvement of DnaE, the second replicative DNA polymerase from *Bacillus subtilis*, in DNA mutagenesis. J Biol Chem.

[47] Sanders GM, Dallmann HG, McHenry CS (2010). Reconstitution of the *B. subtilis* replisome with 13 proteins including two distinct replicases. Mol Cell.

[48] Soultanas P (2012). Loading mechanisms of ring helicases at replication origins. Mol Microbiol.

[49] Rannou O, Le Chatelier E, Larson MA, Nouri H, Dalmais B, Laughton C (2013). Functional interplay of DnaE polymerase, DnaG primase and DnaC helicase within a ternary complex, and primase to polymerase hand-off during lagging strand DNA replication in *Bacillus subtilis*. Nucleic Acids Res.

[50] Dickinson JR, Williams AS (1987). The *cdc30* mutation in *Saccharomyces cerevisiae* results in a temperature-sensitive isoenzyme of phosphoglucose isomerase. J Gen Microbiol.

[51] Fornalewicz K, Wieczorek A, Wegrzyn G, Łyżeń R (2017). Silencing of the pentose phosphate pathway genes influences DNA replication in human fibroblasts. Gene..

[52] Konieczna A, Szczepańska A, Sawiuk K, Wegrzyn G, Łyżeń R (2015). Effects of partial silencing of genes coding for enzymes involved in glycolysis and tricarboxylic acid cycle on the enterance of human fibroblasts to the S phase. BMC Cell Biol.

[53] Sprague GFJ (1977). Isolation and characterization of a *Saccharomyces cerevisiae* mutant deficient in pyruvate kinase activity. J Bacteriol.

[54] Wieczorek A, Fornalewicz K, Mocarski Ł, Łyżeń R, Wegrzyn G (2018). Double silencing of relevant genes suggests the existence of the direct link between DNA replication/repair and central carbon metabolism in human fibroblasts. Gene..

[55] Ronai Z (1993). Glycolytic enzymes as DNA binding proteins. Int J Biochem Cell Biol.

[56] Sirover MA (1999). New insights into an old protein: The functional diversity of mammalian glyceraldehyde-3-phosphate dehydrogenase. Biochim Biophys Acta.

[57] Sirover MA (2011). On the functional diversity of glyceraldehyde-3-phosphate dehydrogenase: Biochemical mechanisms and regulatory control. Biochim Biophys Acta.

[58] Kim J-W, Dang CV (2005). Multifaceted roles of glycolytic enzymes. Trends Biochem Sci.

[59] Konieczna A, Szczepańska A, Sawiuk K, Łyżeń R, Wegrzyn G (2015). Enzymes of the central carbon metabolism: are they linkers between transcription, DNA replication, and carcinogenesis?. Med Hypotheses.

[60] Boukouris AE, Zervopoulos SD, Michelakis ED (2016). Metabolic enzymes moonlighting in the nucleus: metabolic regulation of gene transcription. Trends Biochem Sci.

[61] Lu Z, Hunter T (2018). Metabolic kinases moonlighting as protein kinases. Trends Biochem Sci.

[62] Snaebjornsson MT, Schulze A (2018). Non-canonical functions of enzymes facilitate cross-talk between cell metabolic and regulatory pathways. Exp Mol Med.

[63] Cai L, Sutter BM, Li B, Tu BP (2011). Acetyl-CoA induces cell growth and proliferation by promoting the acetylation of histones at growth genes. Mol Cell.

[64] Sutendra G, Kinnaird A, Dromparis P, Paulin R, Stenson TH, Haromy A (2014). A nuclear pyruvate dehydrogenase complex is important for the generation of Acetyl-CoA and histone acetylation. Cell..

[65] Wellen HG, Sachdeva UM, Bui TV, Cross JR, Thompson CB (2009). ATP-citrate lyase links cellular metabolism to histone acetylation. Science..

[66] Zheng L, Roeder RG, Luo Y (2003). S phase activation of the histone H2B promoter by OCA-S, a coactivator complex that contains GAPDH as a key component. Cell..

[67] Dai RP, Yu FX, Goh SR, Chng HW, Tan YL, Fu JL (2008). Histone 2B (H2B) expression is confined to a proper NAD+/NADH redox status. J Biol Chem.

[68] Ma R, Wu Y, Zhai Y, Hu B, Ma W, Yang W (2019). Exogenous pyruvate represses histone gene expression and inhibits cancer cell proliferation via the NAMPT–NAD+–SIRT1 pathway. Nucleic Acids Res.

[69] Li X, Qian X, Jiang H, Xia Y, Zheng Y, Li J (2018). Nuclear PGK1 alleviates ADP-dependent inhibition of CDC7 to promote DNA replication. Mol Cell.

[70] Grosse F, Nasheuer H-P, Scholtissek S, Schomburg U (1986). Lactate dehydrogenase and glyceraldehyde-phosphate dehydrogenase are single-stranded DNA-binding proteins that affect the DNA-polymerase-α–primase complex. Eur J Biochem.

[71] Popanda O, Fox G, Thielmann HW (1998). Modulation of DNA polymerases alpha, delta and epsilon by lactate dehydrogenase and 3-phosphoglycerat kinase. Biochim Biophys Acta.

[72] Jindal HK, Vishwanatha JK (1990). Functional identity of a primer recognition protein as phosphoglycerate kinase. J Biol Chem.

[73] Schormann N, Hayden KL, Lee P, Banerjee S, Chattopadhyay D (2019). An overview of structure, function, and regulation of pyruvate kinases. Protein Sci.

[74] Suzuki K, Ito S, Shimizu-Ibuka A, Sakai H (2008). Crystal structure of pyruvate kinase from *Geobacillus stearothermophilus*. J Biochem.

[75] Morgan HP, Zhong W, McNae IW, Michels PAM, Fothergill-Gilmore LA, Walkinshaw MD (2014). Structures of pyruvate kinases display evolutionarily divergent allosteric strategies. R Soc Open Sci.

[76] Nicolas P, Mäder U, Dervyn E, Rochat T, Leduc A, Pigeonneau N (2012). Condition-dependent transcriptome reveals high-level regulatory architecture in *Bacillus subtilis*. Science..

[77] Muntel J, Fromion V, Goelzer A, Maab S, Mäder U, Büttner K (2014). Comprehensive absolute quantification of the cytosolic proteome of *Bacillus subtilis* by data independent, parallel fragmentation in liquid chromatographis/mass spectrometry. Mol Cell Proteomics.

[78] Sakai H (2004). Possible structure and function of the extra C-terminal sequence of pyruvate kinase from *Bacillus stearothermophilus*. J Biochem.

[79] Nguyen CC, Saier MH (1995). Phylogenetic analysis of the putative phosphorylation domain in the pyruvate kinase of *Bacillus stearothermophilus*. Res Microbiol.

[80] Alpert CA, Frank R, Stüber K, Deutscher J, Hengstenberg W (1985). Phosphoenolpyruvate-dependent protein kinase enzyme I of *Streptococcus faecalis*: purification and properties of the enzyme and characterization of its active center. Biochemistry..

[81] Teplyakov A, Lim K, Zhu PP, Kapadia G, Chen CCH, Schwartz J (2006). Structure of phosphorylated enzyme I, the phosphoenolpyruvate:sugar phosphotransferase system sugar translocation signal protein. Proc Natl Aca Sci USA.

[82] Goss NH, Evans CT, Wood HG (1980). Pyruvate phosphate dikinase: sequence of the histidyl peptide, the pyrophosphoryl and phosphoryl carrier. Biochemistry..

[83] Herzberg O, Chen CC, Kapadia G, McGuire M, Carroll LJ, Noh SJ (1996). Swiveling-domain mechanism for enzymatic phosphotransfer between remote reaction sites. Proc Natl Aca Sci USA.

[84] Tolentino R, Chastain C, Burnell J (2013). Identification of the amino acid involved in the regulation of bacterial pyruvate, orthophosphate dikinase and phosphoenolpyruvate synthetase. Adv Biol Chem.

[85] Burnell JN, Chastain CJ (2006). Cloning and expression of maize-leaf pyruvate, Pi dikinase regulatory protein gene. Biochem Biophys Res Commun.

[86] Burnell JN (2010). Cloning and characterization of Escherichia coli DUF299: a bifunctional ADP-dependent kinase - P_i_-dependent pyrophosphorylase from bacteria. BMC Biochem.

[87] Burnell JN, Hatch MD (1984). Regulation of C_4_ photosynthesis: Identification of a catalycally important histidine residue and its role in the regulation of pyruvate. Pi dikinase Arch Biochem Biophys.

[88] Eymann C, Dreisbach A, Albrecht D, Bernhardt J, Becher D, Gentner S (2004). A comprehensive proteome map of growing *Bacillus subtilis* cells. Proteomics..

[89] Mäder U, Schmeisky AG, Flórez LA, Stülke J (2012). SubtiWiki--a comprehensive community resource for the model organism Bacillus subtilis. Nucleic Acids Res.

[90] Reiland S, Messerli G, Baerenfaller K, Gerrits B, Endler A, Grossmann J (2009). Large-scale Arabidopsis phosphoproteome profiling reveals novel chloroplast kinase substrates and phosphorylation networks. Plant Physiol.

[91] Pisithkul T, Patel NM, Amador-Noguez D (2015). Post-translational modifications as key regulators of bacterial metabolic fluxes. Curr Opin Microbiol.

[92] Brunk E, Chang RL, Xia J, Hefzi H, Yurkovich JT, Kim D (2018). Characterizing posttranslational modifications in prokaryotic metabolism using a multiscale workflow. Proc Natl Aca Sci USA.

[93] Bollenbach TJ, Mesecar AD, Nowak T (1999). Role of lysine 240 in the mechanism of yeast pyruvate kinase catalysis. Biochemistry..

[94] Stuani L, Lechaplais C, Salminen AV, Sérugens B, Durot M, Castelli V (2014). Novel metabolic features in *Acinetobacter baylyi* ADP1 revealed by a multiomics approach. Metabolomics..

[95] Dubois T, Krzewinski F, Yamakawa N, Lemy C, Hamiot A, Brunet L (2020). Genes encode an original legionaminic acid pathway required for crust assembly in *Bacillus subtilis*. mBio..

[96] Washington TA, Smith JL, Grossman AD (2017). Genetic networks controlled by the bacterial replication initiator and transcription factor DnaA in *Bacillus subtilis*. Mol Microbiol.

[97] Morigen OI, Skarstad K (2009). Growth rate dependent numbers of SeqA structures organize the multiple replication forks in rapidly growing *Escherichia coli*. Genes Cells.

[98] Gao S, Bao J, Gu X, Xin X, Chen C, Ryu DDY (2008). Substrate promiscuity of pyruvate kinase on various deoxynucleoside diphosphates for synthesis of deoxynucleoside triphosphates. Enzyme Microb Technol.

[99] Meile J-C, Wu LJ, Ehrlich SD, Errington J, Noirot P (2006). Systematic localisation of proteins fused to the green fluorescent protein inBacillus subtilis: Identification of new proteins at the DNA replication factory. Proteomics..

[100] Bruck I, O'Donnell M (2000). The DNA replication machine of a gram-positive organism. J Biol Chem.

[101] Chuang C, Prasanth KR, Nagy PD (2017). The glycolytic pyruvate kinase is recruited directly into the viral replicase complex to generate ATP for RNA synthesis. Cell Host Microbe.

[102] Pancholi V, Chhatwal GS (2003). Housekeeping enzymes as virulence factors for pathogens. Int J Med Microbiol.

[103] Tu BP, Mohler RE, Liu JC, Dombek KM, Young ET, Synovec RE (2007). Cyclic changes in metabolic state during the life of a yeast cell. Proc Natl Aca Sci USA.

[104] Ewald JC, Kuehne A, Zamboni N, Skotheim JM (2016). The yeast cyclin-dependent kinase routes carbon fluxes to fuel cell cycle progression. Mol Cell.

[105] Hartl J, Kiefer P, Kaczmarczyk A, Mittelviefhaus M, Meyer F, Vonderach T (2020). Untargeted metabolomics links glutathione to bacterial cell cycle progression. Nat Metab.

[106] Lee J-B, Hite RK, Hamdan SM, Xie SX, Richardson CC, van Oijen AM (2006). DNA primase acts as a molecular break in DNA replication. Nature..

[107] Tanner NA, Hamdan SM, Jergic S, Schaeffer PM, Dixon NE, van Oijen AM (2008). Single-molecule studies of fork dynamics in *Escherichia col*i DNA replication. Nat Struct Mol Biol.

[108] Yao NY, Georgescu RE, Finkelstein J, O'Donnell ME (2009). Single-molecule analysis reveals that the lagging strand increases replisome processivity but slows replication fork progression. Proc Natl Aca Sci USA.

[109] Georgescu RE, Yao N, Indiani C, Yurieva O, O'Donnell ME (2014). Replisome mechanics: lagging strand events that influence speed and processivity. Nucleic Acids Res.

[110] Rodgers K, McVey M (2016). Error-Prone repair of DNA double-strand breaks. J Cell Physiol.

[111] Loeb LA (2011). Human cancers express mutator phenotypes: origin, consequences and targeting. Nat Rev Cancer.

[112] Macheret M, Halazonetis TD (2015). DNA replication stress as a hallmark of cancer. Annu Rev Pathol Mech Dis.

[113] Oskam L, Hillenga DJ, Venema G, Bron S (1991). The large *Bacillus* plasmid pTB19 contains two integrated rolling-circle plasmids carrying mobilization functions. Plasmid..

[114] Magill NG, Setlow P (1992). Properties of purified sporlets produced by spoII mutants of Bacillus subtilis. J Bacteriol Am Soc Microbiol.

[115] Séror SJ, Casarégola S, Vannier F, Zouari N, Dahl M, Boye E (1994). A mutant cysteinyl-tRNA synthetase affecting timing of chromosomal replication initiation in *B. subtilis* and conferring resistance to a protein kinase C inhibitor. EMBO J.

[116] Thomas M, Stuani L, Darii E, Lechaplais C, Pateau E, Tabet J-C (2019). De novo structure determination of 3-((3-aminopropyl)amino)- 4-hydroxybenzoic acid, a novel and abundant metabolite in *Acinetobacter baylyi* ADP1. Metabolomics..

[117] Chambers MC, Maclean B, Burke R, Amode D, Ruderman DL, Neumann S (2012). A cross-platform toolkit for mass spectrometry and proteomics. Nat Biotechnol.

[118] Smith CA, Want EJ, OMaille G, Abagyan R, Siuzdak G. (2006). XCMS: Processing mass spectrometry data for metabolite profiling using nonlinear peak alignment, matching, and identification. Anal Chem.

[119] Tautenhahn R, Böttcher C, Neumann S (2008). Highly sensitive feature detection for high resolution LC/MS. BMC Bioinformatics.

[120] Gaude E, Chignola F, Spiliotopoulos D, Spitaleri A, Ghitti M, Garcia-Manteiga J (2013). *muma*, an R package for metabolomics univariate and multivariate statistical analysis. Curr Metabolomics.

[121] Lê S, Josse J, Husson F (2008). FactoMineR: An R package for multivariate analysis. J Stat Softw.

[122] Haug (2020). MetaboLights : a resource evolving in response to the needs of its scientific community. Nucleic Acids Res.

