# Supplementary figures and images for: Pyruvate kinase, a metabolic sensor powering glycolysis, drives the metabolic control of DNA replication
Source: BMC Biol. 2022 Apr 13;20:87. doi: 10.1186/s12915-022-01278-3 (PMC9009071; doi:10.1186/s12915-022-01278-3)

## Slide 1
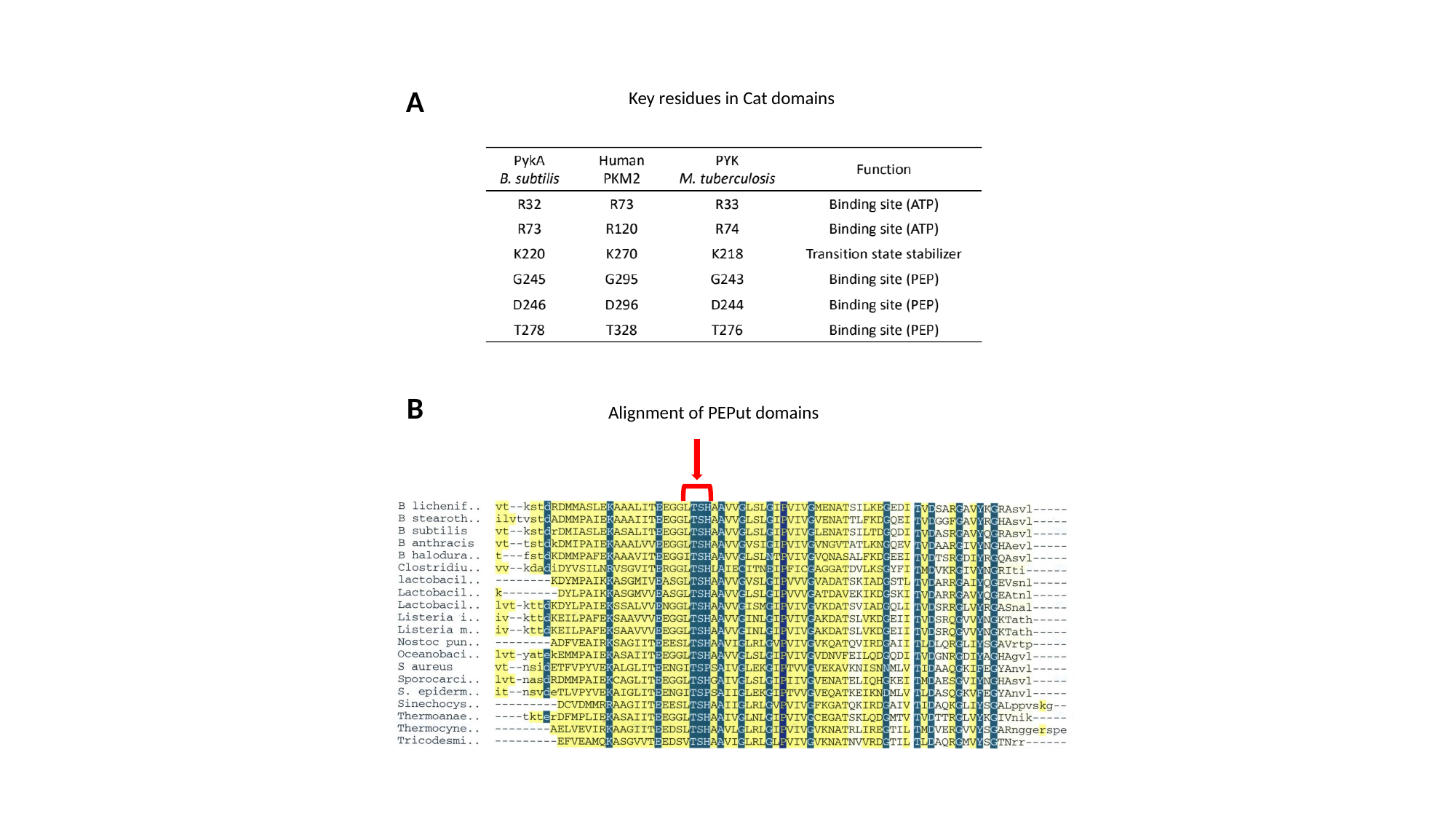

Key residues in Cat domains
A
B
Alignment of PEPut domains

Supplement: Supplementary file 1 — Additional file 1: Fig. S1. Key amino-acids of the Cat and PEPut domains of PykA. A. Cat domain analysis. Clustalw and Chimera analysis of the pyruvate kinase of B. subtilis (PykA), human cells (PKM2) and Mycobacterium tuberculosis (PYK) identified key amino acids of the catalytic site of the B. subtilis protein. B. PEPut domain analysis. Alignment of the PEPut domain of PykA to related domains of various metabolic enzymes. The red arrow highlights the conserved LTSH motif (coordinates 536-539). Fig. S2. Effect of Cat and PEPut mutations on growth in MC. Wild-type and pykA mutants were first grown over-night in MC supplemented with antibiotic when appropriate. Upon saturation, cultures were diluted 1000-fold in the same medium without antibiotic and growth was monitored spectrophometrically. Left panel: Analysis of catalytic mutants (pykAΔcat, pykAR32A, pykAR73A, pykAK220A, pykAGD245/6AA, pykAT278A, pykAJP). Right panel: Analysis of PEPut and Cat-PEPut interaction mutants (pykAΔPEP, pykAT>A, pykAS>A, pykAH>A, pykATSH>AAA, pykAT>D, pykAS>D, pykAH>D, pykATSH>DDD, pykAE209A, pykAL536A). Controls: TF8A (wild-type) and ΔpykA. Fig. S3. Analysis of NTP in the metabolome of wild-type and pykAT>D cells. ATP, GTP and CTP were detected in the positive ionization mode. UTP was detected in the negative ionization mode. Note that TTP signals were too low for quantifications. Data correspond to 3 independent extractions (solid cultures).*, p > 0.05 ; **, p < 0.05 (Welch's T-test). Values in bold indicate the fold change for each metabolite (WT vs pykAT>D). Fig. S4. LC/MS analysis of legionaminic acid in the metabolome. A. Extracted ion chromatogram (EIC) corresponds to the deprotonated molecule [M-H]- at m/z 333.1303 (5 ppm accuracy). B. Zoom on the mass spectrum of legionaminic acid in the negative mode. C. Collision Induced dissociation (CID) spectrum of legionaminic acid in the negative mode at 22% Normalized Collision Energy (NCE). D. CID spectrum of legionaminic acid in the p [file 12915_2022_1278_MOESM1_ESM.zip › Fig. S1.pptx]

## Slide 1
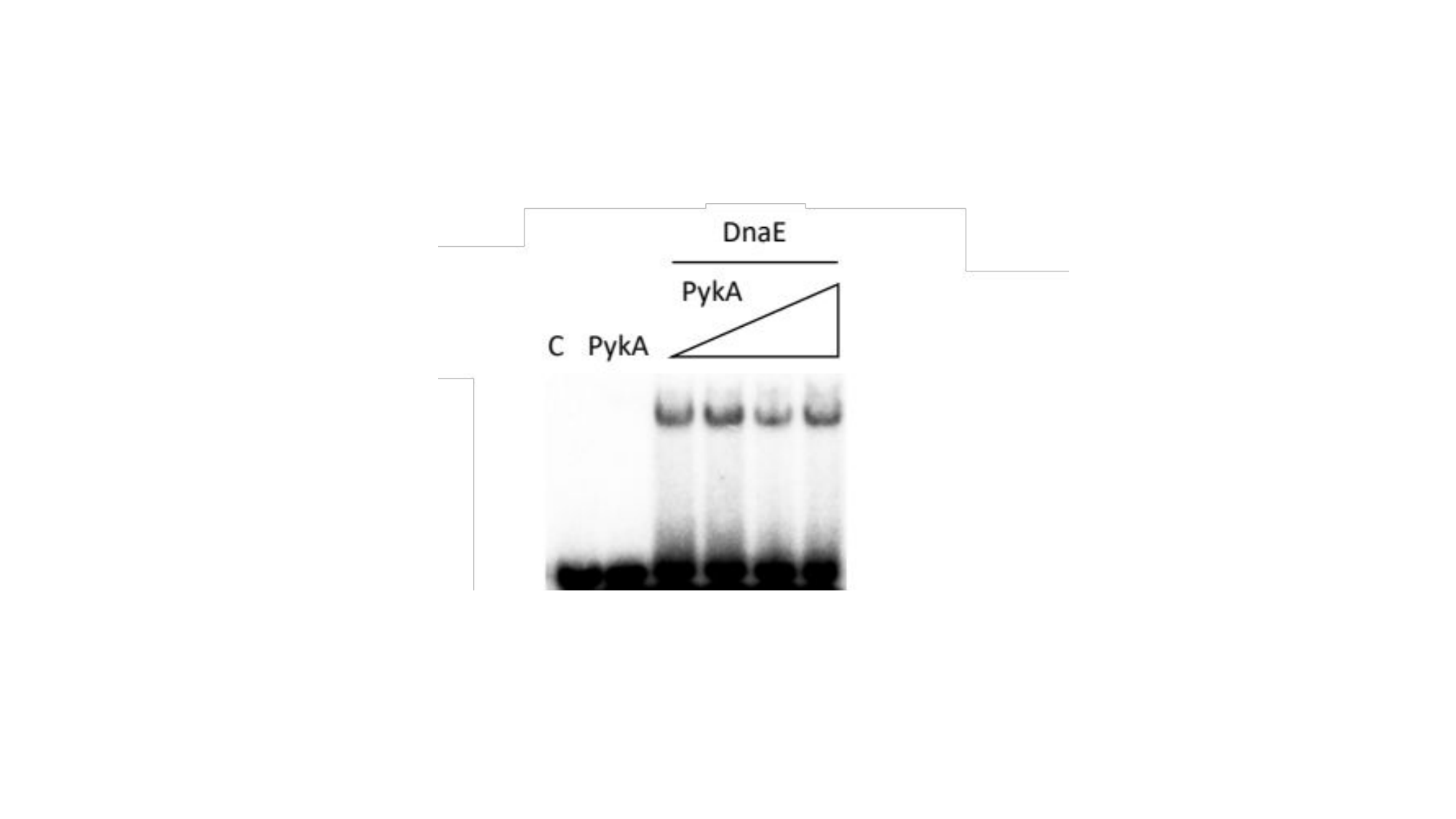

Supplement: Supplementary file 1 — Additional file 1: Fig. S1. Key amino-acids of the Cat and PEPut domains of PykA. A. Cat domain analysis. Clustalw and Chimera analysis of the pyruvate kinase of B. subtilis (PykA), human cells (PKM2) and Mycobacterium tuberculosis (PYK) identified key amino acids of the catalytic site of the B. subtilis protein. B. PEPut domain analysis. Alignment of the PEPut domain of PykA to related domains of various metabolic enzymes. The red arrow highlights the conserved LTSH motif (coordinates 536-539). Fig. S2. Effect of Cat and PEPut mutations on growth in MC. Wild-type and pykA mutants were first grown over-night in MC supplemented with antibiotic when appropriate. Upon saturation, cultures were diluted 1000-fold in the same medium without antibiotic and growth was monitored spectrophometrically. Left panel: Analysis of catalytic mutants (pykAΔcat, pykAR32A, pykAR73A, pykAK220A, pykAGD245/6AA, pykAT278A, pykAJP). Right panel: Analysis of PEPut and Cat-PEPut interaction mutants (pykAΔPEP, pykAT>A, pykAS>A, pykAH>A, pykATSH>AAA, pykAT>D, pykAS>D, pykAH>D, pykATSH>DDD, pykAE209A, pykAL536A). Controls: TF8A (wild-type) and ΔpykA. Fig. S3. Analysis of NTP in the metabolome of wild-type and pykAT>D cells. ATP, GTP and CTP were detected in the positive ionization mode. UTP was detected in the negative ionization mode. Note that TTP signals were too low for quantifications. Data correspond to 3 independent extractions (solid cultures).*, p > 0.05 ; **, p < 0.05 (Welch's T-test). Values in bold indicate the fold change for each metabolite (WT vs pykAT>D). Fig. S4. LC/MS analysis of legionaminic acid in the metabolome. A. Extracted ion chromatogram (EIC) corresponds to the deprotonated molecule [M-H]- at m/z 333.1303 (5 ppm accuracy). B. Zoom on the mass spectrum of legionaminic acid in the negative mode. C. Collision Induced dissociation (CID) spectrum of legionaminic acid in the negative mode at 22% Normalized Collision Energy (NCE). D. CID spectrum of legionaminic acid in the p [file 12915_2022_1278_MOESM1_ESM.zip › Fig. S10.pptx]

## Slide 1
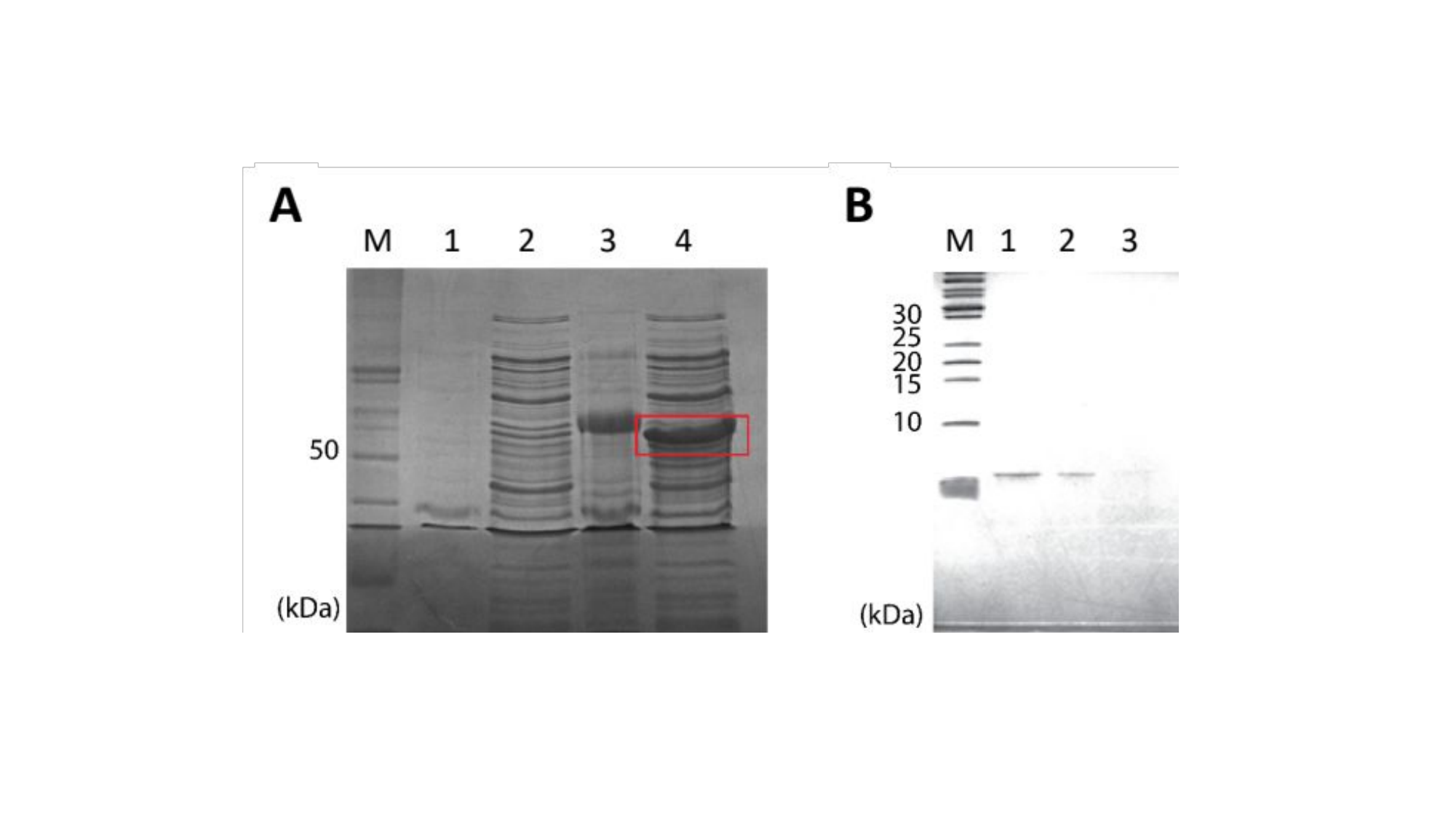

Supplement: Supplementary file 1 — Additional file 1: Fig. S1. Key amino-acids of the Cat and PEPut domains of PykA. A. Cat domain analysis. Clustalw and Chimera analysis of the pyruvate kinase of B. subtilis (PykA), human cells (PKM2) and Mycobacterium tuberculosis (PYK) identified key amino acids of the catalytic site of the B. subtilis protein. B. PEPut domain analysis. Alignment of the PEPut domain of PykA to related domains of various metabolic enzymes. The red arrow highlights the conserved LTSH motif (coordinates 536-539). Fig. S2. Effect of Cat and PEPut mutations on growth in MC. Wild-type and pykA mutants were first grown over-night in MC supplemented with antibiotic when appropriate. Upon saturation, cultures were diluted 1000-fold in the same medium without antibiotic and growth was monitored spectrophometrically. Left panel: Analysis of catalytic mutants (pykAΔcat, pykAR32A, pykAR73A, pykAK220A, pykAGD245/6AA, pykAT278A, pykAJP). Right panel: Analysis of PEPut and Cat-PEPut interaction mutants (pykAΔPEP, pykAT>A, pykAS>A, pykAH>A, pykATSH>AAA, pykAT>D, pykAS>D, pykAH>D, pykATSH>DDD, pykAE209A, pykAL536A). Controls: TF8A (wild-type) and ΔpykA. Fig. S3. Analysis of NTP in the metabolome of wild-type and pykAT>D cells. ATP, GTP and CTP were detected in the positive ionization mode. UTP was detected in the negative ionization mode. Note that TTP signals were too low for quantifications. Data correspond to 3 independent extractions (solid cultures).*, p > 0.05 ; **, p < 0.05 (Welch's T-test). Values in bold indicate the fold change for each metabolite (WT vs pykAT>D). Fig. S4. LC/MS analysis of legionaminic acid in the metabolome. A. Extracted ion chromatogram (EIC) corresponds to the deprotonated molecule [M-H]- at m/z 333.1303 (5 ppm accuracy). B. Zoom on the mass spectrum of legionaminic acid in the negative mode. C. Collision Induced dissociation (CID) spectrum of legionaminic acid in the negative mode at 22% Normalized Collision Energy (NCE). D. CID spectrum of legionaminic acid in the p [file 12915_2022_1278_MOESM1_ESM.zip › Fig. S11.pptx]

## Slide 1
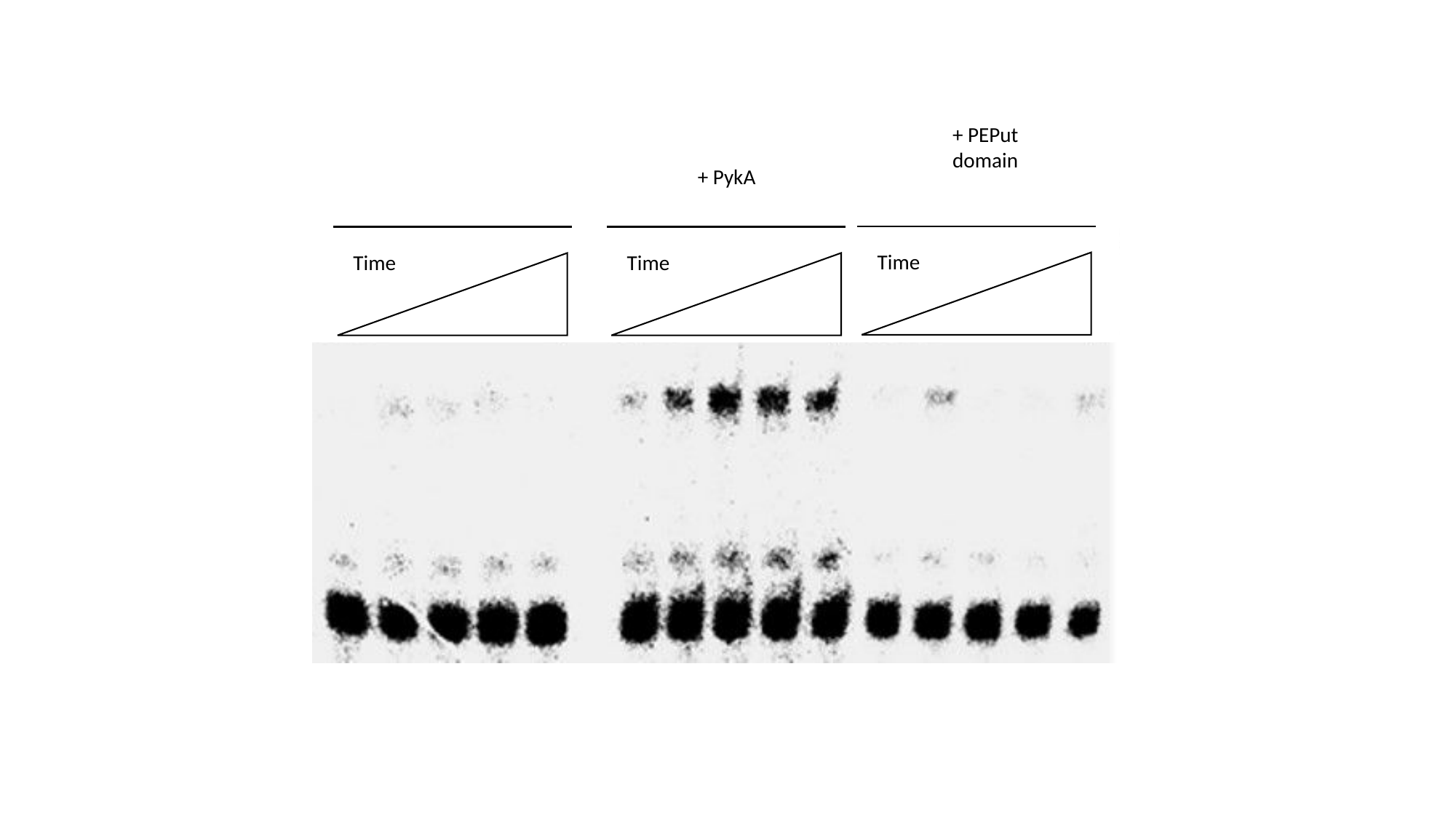

+ PEPut
domain
+ PykA
Time
Time
Time

Supplement: Supplementary file 1 — Additional file 1: Fig. S1. Key amino-acids of the Cat and PEPut domains of PykA. A. Cat domain analysis. Clustalw and Chimera analysis of the pyruvate kinase of B. subtilis (PykA), human cells (PKM2) and Mycobacterium tuberculosis (PYK) identified key amino acids of the catalytic site of the B. subtilis protein. B. PEPut domain analysis. Alignment of the PEPut domain of PykA to related domains of various metabolic enzymes. The red arrow highlights the conserved LTSH motif (coordinates 536-539). Fig. S2. Effect of Cat and PEPut mutations on growth in MC. Wild-type and pykA mutants were first grown over-night in MC supplemented with antibiotic when appropriate. Upon saturation, cultures were diluted 1000-fold in the same medium without antibiotic and growth was monitored spectrophometrically. Left panel: Analysis of catalytic mutants (pykAΔcat, pykAR32A, pykAR73A, pykAK220A, pykAGD245/6AA, pykAT278A, pykAJP). Right panel: Analysis of PEPut and Cat-PEPut interaction mutants (pykAΔPEP, pykAT>A, pykAS>A, pykAH>A, pykATSH>AAA, pykAT>D, pykAS>D, pykAH>D, pykATSH>DDD, pykAE209A, pykAL536A). Controls: TF8A (wild-type) and ΔpykA. Fig. S3. Analysis of NTP in the metabolome of wild-type and pykAT>D cells. ATP, GTP and CTP were detected in the positive ionization mode. UTP was detected in the negative ionization mode. Note that TTP signals were too low for quantifications. Data correspond to 3 independent extractions (solid cultures).*, p > 0.05 ; **, p < 0.05 (Welch's T-test). Values in bold indicate the fold change for each metabolite (WT vs pykAT>D). Fig. S4. LC/MS analysis of legionaminic acid in the metabolome. A. Extracted ion chromatogram (EIC) corresponds to the deprotonated molecule [M-H]- at m/z 333.1303 (5 ppm accuracy). B. Zoom on the mass spectrum of legionaminic acid in the negative mode. C. Collision Induced dissociation (CID) spectrum of legionaminic acid in the negative mode at 22% Normalized Collision Energy (NCE). D. CID spectrum of legionaminic acid in the p [file 12915_2022_1278_MOESM1_ESM.zip › Fig. S12.pptx]

## Slide 1
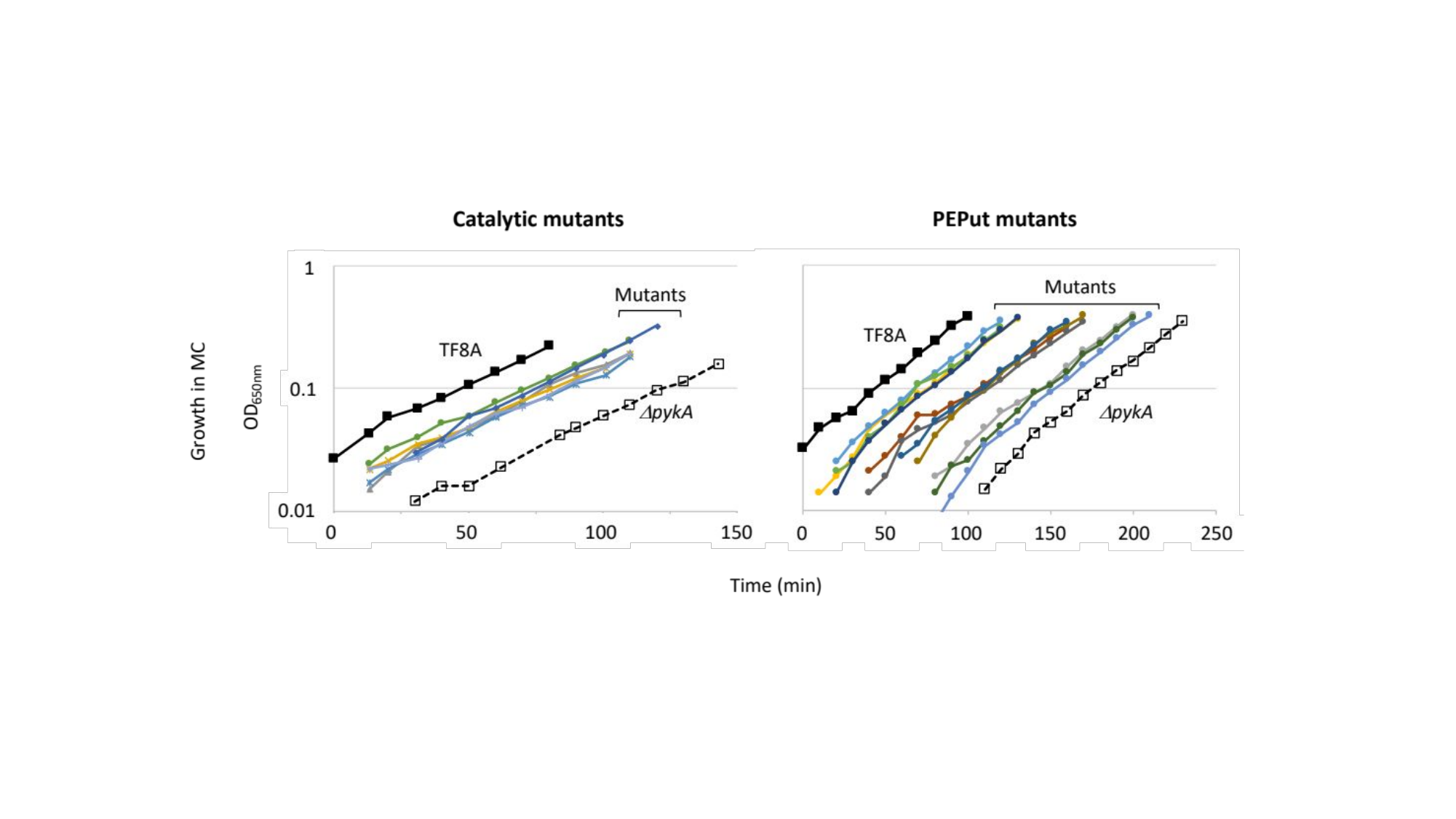

Supplement: Supplementary file 1 — Additional file 1: Fig. S1. Key amino-acids of the Cat and PEPut domains of PykA. A. Cat domain analysis. Clustalw and Chimera analysis of the pyruvate kinase of B. subtilis (PykA), human cells (PKM2) and Mycobacterium tuberculosis (PYK) identified key amino acids of the catalytic site of the B. subtilis protein. B. PEPut domain analysis. Alignment of the PEPut domain of PykA to related domains of various metabolic enzymes. The red arrow highlights the conserved LTSH motif (coordinates 536-539). Fig. S2. Effect of Cat and PEPut mutations on growth in MC. Wild-type and pykA mutants were first grown over-night in MC supplemented with antibiotic when appropriate. Upon saturation, cultures were diluted 1000-fold in the same medium without antibiotic and growth was monitored spectrophometrically. Left panel: Analysis of catalytic mutants (pykAΔcat, pykAR32A, pykAR73A, pykAK220A, pykAGD245/6AA, pykAT278A, pykAJP). Right panel: Analysis of PEPut and Cat-PEPut interaction mutants (pykAΔPEP, pykAT>A, pykAS>A, pykAH>A, pykATSH>AAA, pykAT>D, pykAS>D, pykAH>D, pykATSH>DDD, pykAE209A, pykAL536A). Controls: TF8A (wild-type) and ΔpykA. Fig. S3. Analysis of NTP in the metabolome of wild-type and pykAT>D cells. ATP, GTP and CTP were detected in the positive ionization mode. UTP was detected in the negative ionization mode. Note that TTP signals were too low for quantifications. Data correspond to 3 independent extractions (solid cultures).*, p > 0.05 ; **, p < 0.05 (Welch's T-test). Values in bold indicate the fold change for each metabolite (WT vs pykAT>D). Fig. S4. LC/MS analysis of legionaminic acid in the metabolome. A. Extracted ion chromatogram (EIC) corresponds to the deprotonated molecule [M-H]- at m/z 333.1303 (5 ppm accuracy). B. Zoom on the mass spectrum of legionaminic acid in the negative mode. C. Collision Induced dissociation (CID) spectrum of legionaminic acid in the negative mode at 22% Normalized Collision Energy (NCE). D. CID spectrum of legionaminic acid in the p [file 12915_2022_1278_MOESM1_ESM.zip › Fig. S2.pptx]

## Slide 1
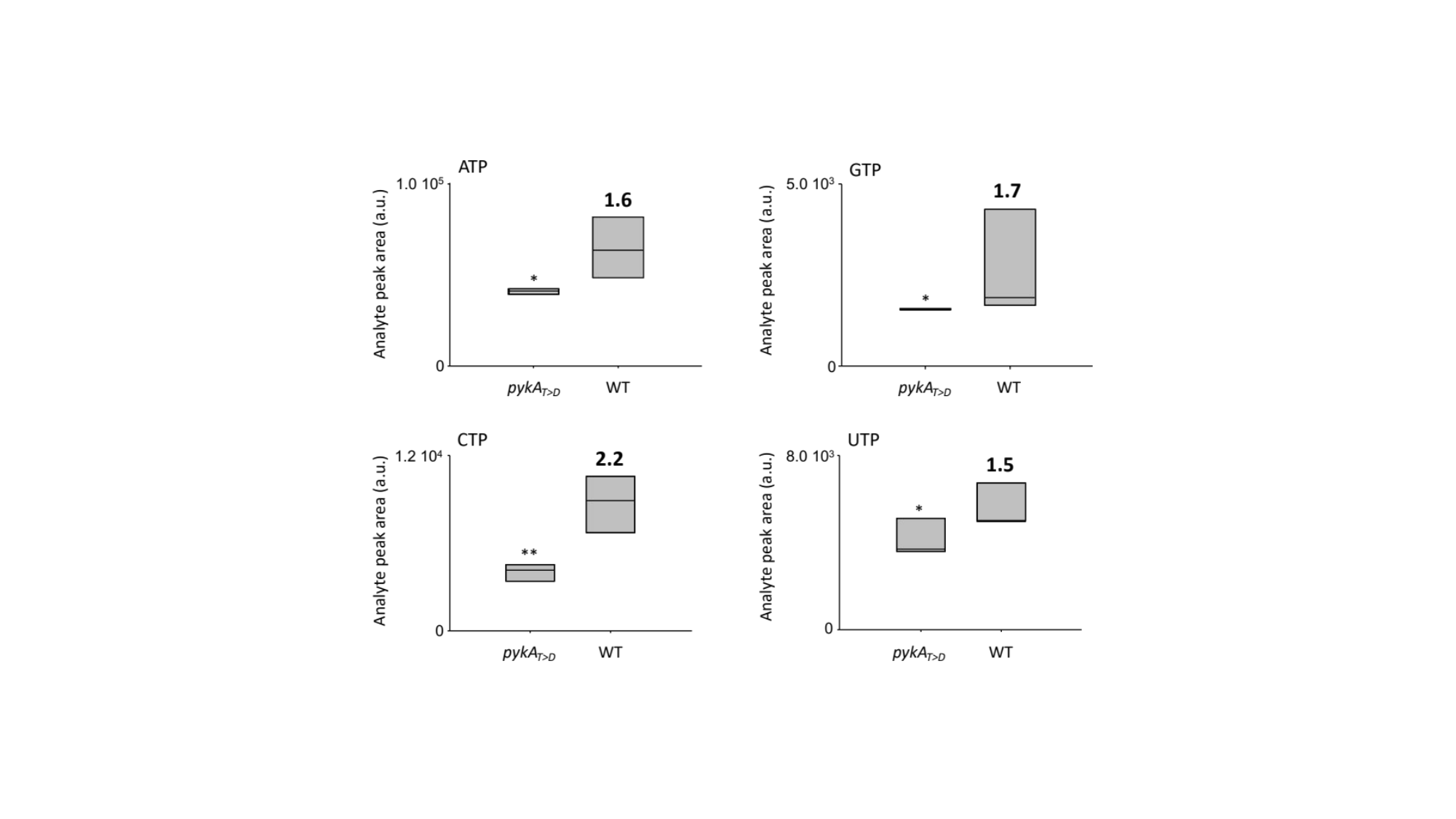

Supplement: Supplementary file 1 — Additional file 1: Fig. S1. Key amino-acids of the Cat and PEPut domains of PykA. A. Cat domain analysis. Clustalw and Chimera analysis of the pyruvate kinase of B. subtilis (PykA), human cells (PKM2) and Mycobacterium tuberculosis (PYK) identified key amino acids of the catalytic site of the B. subtilis protein. B. PEPut domain analysis. Alignment of the PEPut domain of PykA to related domains of various metabolic enzymes. The red arrow highlights the conserved LTSH motif (coordinates 536-539). Fig. S2. Effect of Cat and PEPut mutations on growth in MC. Wild-type and pykA mutants were first grown over-night in MC supplemented with antibiotic when appropriate. Upon saturation, cultures were diluted 1000-fold in the same medium without antibiotic and growth was monitored spectrophometrically. Left panel: Analysis of catalytic mutants (pykAΔcat, pykAR32A, pykAR73A, pykAK220A, pykAGD245/6AA, pykAT278A, pykAJP). Right panel: Analysis of PEPut and Cat-PEPut interaction mutants (pykAΔPEP, pykAT>A, pykAS>A, pykAH>A, pykATSH>AAA, pykAT>D, pykAS>D, pykAH>D, pykATSH>DDD, pykAE209A, pykAL536A). Controls: TF8A (wild-type) and ΔpykA. Fig. S3. Analysis of NTP in the metabolome of wild-type and pykAT>D cells. ATP, GTP and CTP were detected in the positive ionization mode. UTP was detected in the negative ionization mode. Note that TTP signals were too low for quantifications. Data correspond to 3 independent extractions (solid cultures).*, p > 0.05 ; **, p < 0.05 (Welch's T-test). Values in bold indicate the fold change for each metabolite (WT vs pykAT>D). Fig. S4. LC/MS analysis of legionaminic acid in the metabolome. A. Extracted ion chromatogram (EIC) corresponds to the deprotonated molecule [M-H]- at m/z 333.1303 (5 ppm accuracy). B. Zoom on the mass spectrum of legionaminic acid in the negative mode. C. Collision Induced dissociation (CID) spectrum of legionaminic acid in the negative mode at 22% Normalized Collision Energy (NCE). D. CID spectrum of legionaminic acid in the p [file 12915_2022_1278_MOESM1_ESM.zip › Fig. S3.pptx]

## Slide 1
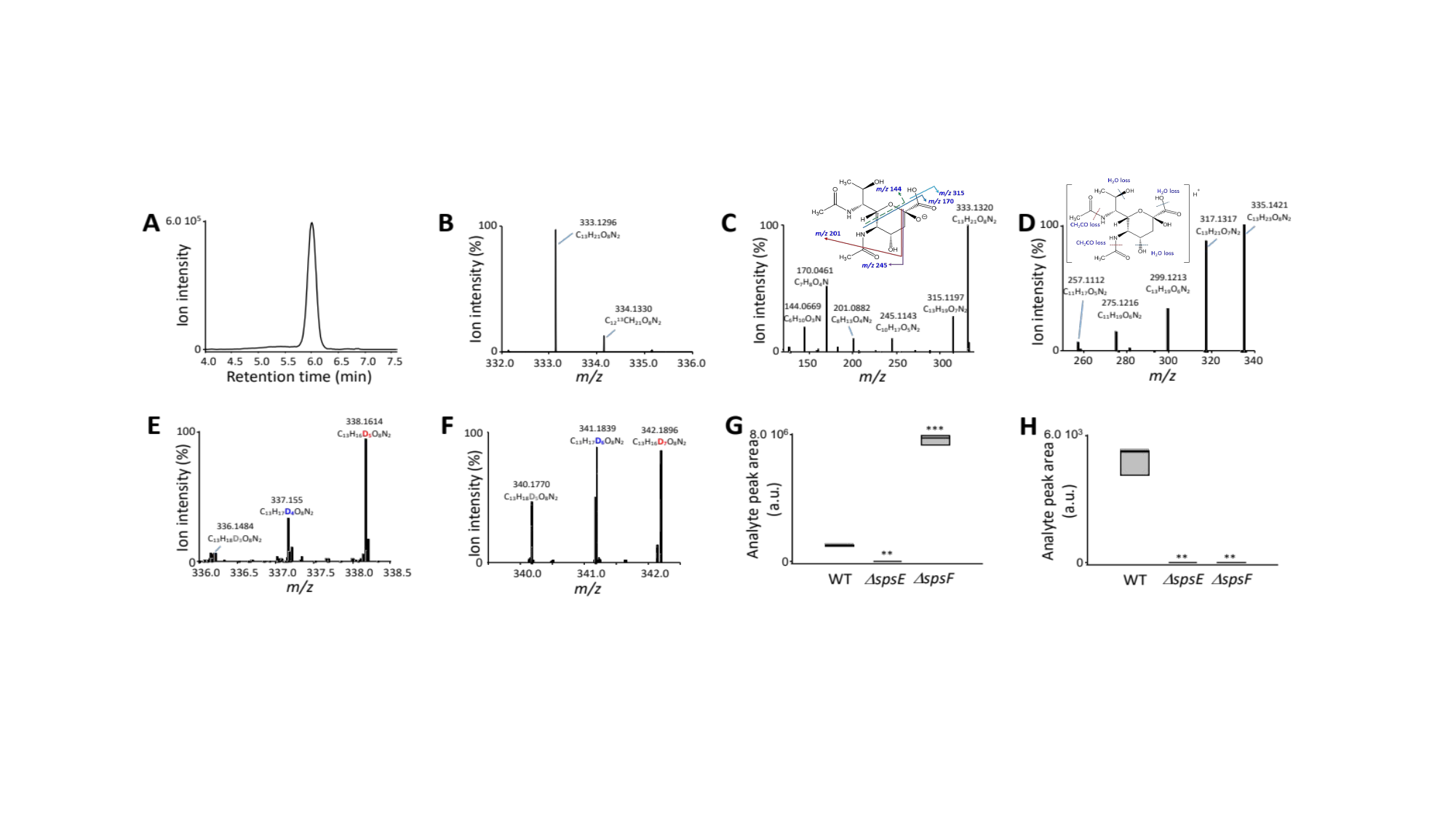

Supplement: Supplementary file 1 — Additional file 1: Fig. S1. Key amino-acids of the Cat and PEPut domains of PykA. A. Cat domain analysis. Clustalw and Chimera analysis of the pyruvate kinase of B. subtilis (PykA), human cells (PKM2) and Mycobacterium tuberculosis (PYK) identified key amino acids of the catalytic site of the B. subtilis protein. B. PEPut domain analysis. Alignment of the PEPut domain of PykA to related domains of various metabolic enzymes. The red arrow highlights the conserved LTSH motif (coordinates 536-539). Fig. S2. Effect of Cat and PEPut mutations on growth in MC. Wild-type and pykA mutants were first grown over-night in MC supplemented with antibiotic when appropriate. Upon saturation, cultures were diluted 1000-fold in the same medium without antibiotic and growth was monitored spectrophometrically. Left panel: Analysis of catalytic mutants (pykAΔcat, pykAR32A, pykAR73A, pykAK220A, pykAGD245/6AA, pykAT278A, pykAJP). Right panel: Analysis of PEPut and Cat-PEPut interaction mutants (pykAΔPEP, pykAT>A, pykAS>A, pykAH>A, pykATSH>AAA, pykAT>D, pykAS>D, pykAH>D, pykATSH>DDD, pykAE209A, pykAL536A). Controls: TF8A (wild-type) and ΔpykA. Fig. S3. Analysis of NTP in the metabolome of wild-type and pykAT>D cells. ATP, GTP and CTP were detected in the positive ionization mode. UTP was detected in the negative ionization mode. Note that TTP signals were too low for quantifications. Data correspond to 3 independent extractions (solid cultures).*, p > 0.05 ; **, p < 0.05 (Welch's T-test). Values in bold indicate the fold change for each metabolite (WT vs pykAT>D). Fig. S4. LC/MS analysis of legionaminic acid in the metabolome. A. Extracted ion chromatogram (EIC) corresponds to the deprotonated molecule [M-H]- at m/z 333.1303 (5 ppm accuracy). B. Zoom on the mass spectrum of legionaminic acid in the negative mode. C. Collision Induced dissociation (CID) spectrum of legionaminic acid in the negative mode at 22% Normalized Collision Energy (NCE). D. CID spectrum of legionaminic acid in the p [file 12915_2022_1278_MOESM1_ESM.zip › Fig. S4.pptx]

## Slide 1
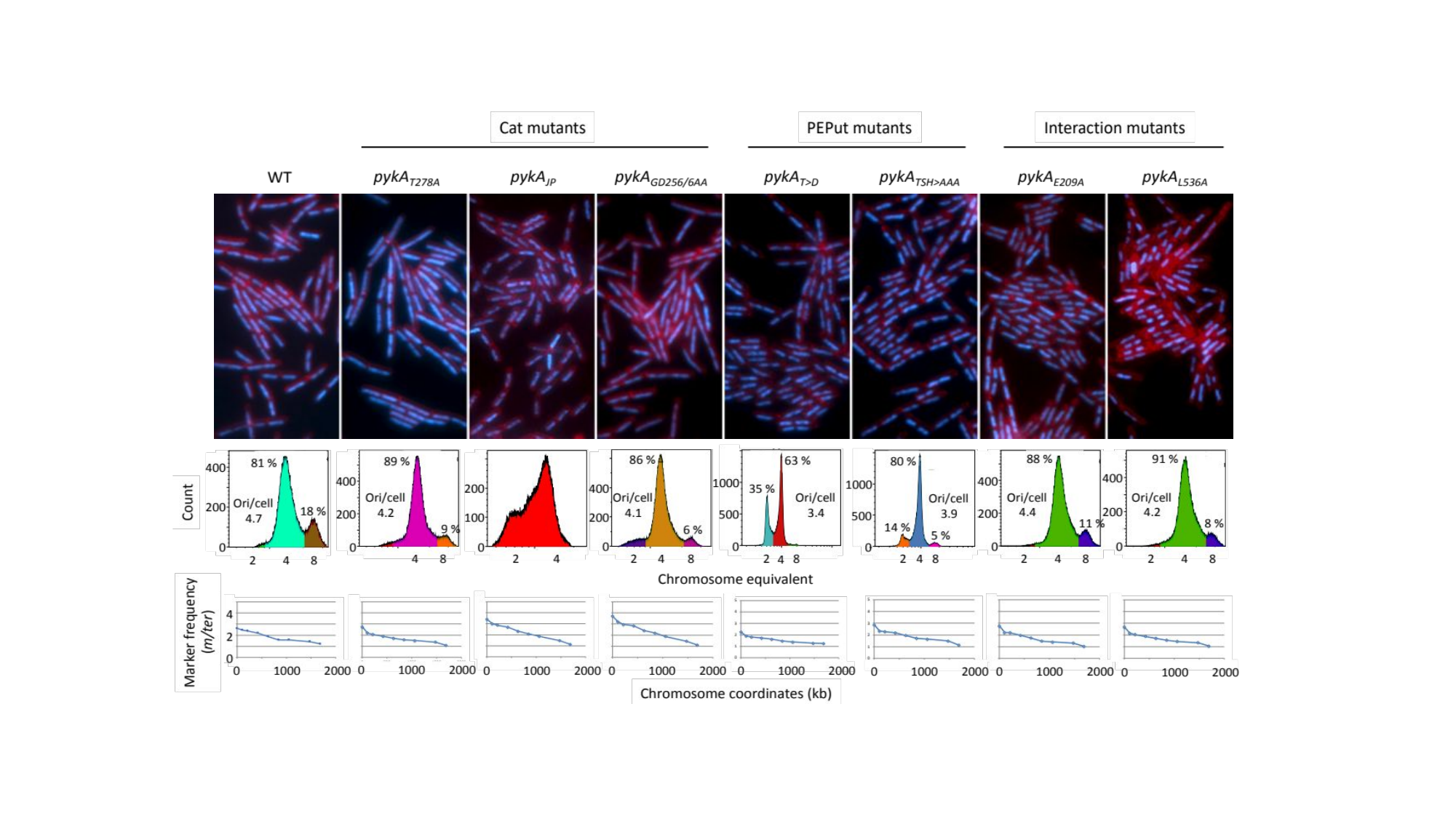

Supplement: Supplementary file 1 — Additional file 1: Fig. S1. Key amino-acids of the Cat and PEPut domains of PykA. A. Cat domain analysis. Clustalw and Chimera analysis of the pyruvate kinase of B. subtilis (PykA), human cells (PKM2) and Mycobacterium tuberculosis (PYK) identified key amino acids of the catalytic site of the B. subtilis protein. B. PEPut domain analysis. Alignment of the PEPut domain of PykA to related domains of various metabolic enzymes. The red arrow highlights the conserved LTSH motif (coordinates 536-539). Fig. S2. Effect of Cat and PEPut mutations on growth in MC. Wild-type and pykA mutants were first grown over-night in MC supplemented with antibiotic when appropriate. Upon saturation, cultures were diluted 1000-fold in the same medium without antibiotic and growth was monitored spectrophometrically. Left panel: Analysis of catalytic mutants (pykAΔcat, pykAR32A, pykAR73A, pykAK220A, pykAGD245/6AA, pykAT278A, pykAJP). Right panel: Analysis of PEPut and Cat-PEPut interaction mutants (pykAΔPEP, pykAT>A, pykAS>A, pykAH>A, pykATSH>AAA, pykAT>D, pykAS>D, pykAH>D, pykATSH>DDD, pykAE209A, pykAL536A). Controls: TF8A (wild-type) and ΔpykA. Fig. S3. Analysis of NTP in the metabolome of wild-type and pykAT>D cells. ATP, GTP and CTP were detected in the positive ionization mode. UTP was detected in the negative ionization mode. Note that TTP signals were too low for quantifications. Data correspond to 3 independent extractions (solid cultures).*, p > 0.05 ; **, p < 0.05 (Welch's T-test). Values in bold indicate the fold change for each metabolite (WT vs pykAT>D). Fig. S4. LC/MS analysis of legionaminic acid in the metabolome. A. Extracted ion chromatogram (EIC) corresponds to the deprotonated molecule [M-H]- at m/z 333.1303 (5 ppm accuracy). B. Zoom on the mass spectrum of legionaminic acid in the negative mode. C. Collision Induced dissociation (CID) spectrum of legionaminic acid in the negative mode at 22% Normalized Collision Energy (NCE). D. CID spectrum of legionaminic acid in the p [file 12915_2022_1278_MOESM1_ESM.zip › Fig. S5.pptx]

## Slide 1
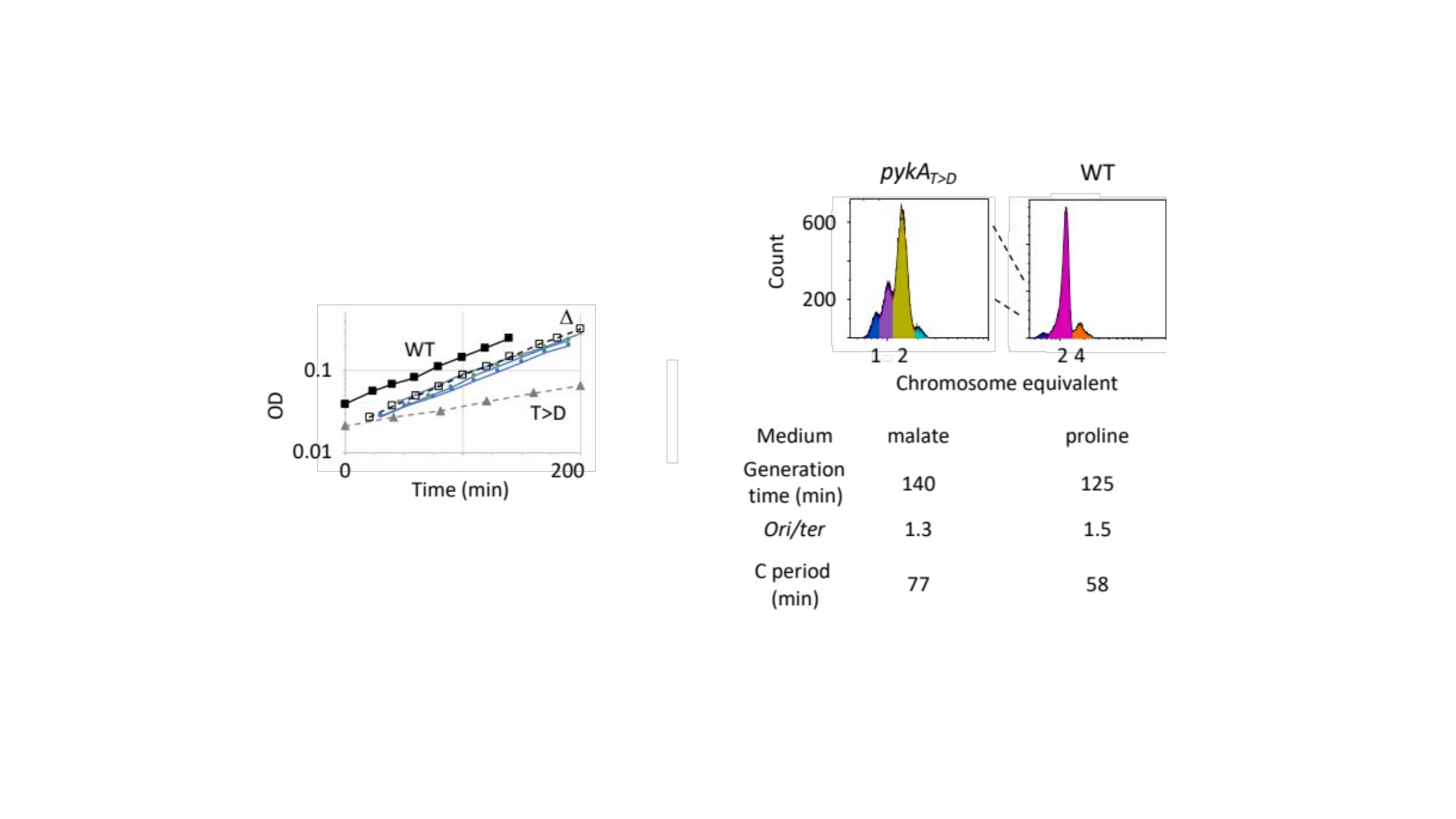

Supplement: Supplementary file 1 — Additional file 1: Fig. S1. Key amino-acids of the Cat and PEPut domains of PykA. A. Cat domain analysis. Clustalw and Chimera analysis of the pyruvate kinase of B. subtilis (PykA), human cells (PKM2) and Mycobacterium tuberculosis (PYK) identified key amino acids of the catalytic site of the B. subtilis protein. B. PEPut domain analysis. Alignment of the PEPut domain of PykA to related domains of various metabolic enzymes. The red arrow highlights the conserved LTSH motif (coordinates 536-539). Fig. S2. Effect of Cat and PEPut mutations on growth in MC. Wild-type and pykA mutants were first grown over-night in MC supplemented with antibiotic when appropriate. Upon saturation, cultures were diluted 1000-fold in the same medium without antibiotic and growth was monitored spectrophometrically. Left panel: Analysis of catalytic mutants (pykAΔcat, pykAR32A, pykAR73A, pykAK220A, pykAGD245/6AA, pykAT278A, pykAJP). Right panel: Analysis of PEPut and Cat-PEPut interaction mutants (pykAΔPEP, pykAT>A, pykAS>A, pykAH>A, pykATSH>AAA, pykAT>D, pykAS>D, pykAH>D, pykATSH>DDD, pykAE209A, pykAL536A). Controls: TF8A (wild-type) and ΔpykA. Fig. S3. Analysis of NTP in the metabolome of wild-type and pykAT>D cells. ATP, GTP and CTP were detected in the positive ionization mode. UTP was detected in the negative ionization mode. Note that TTP signals were too low for quantifications. Data correspond to 3 independent extractions (solid cultures).*, p > 0.05 ; **, p < 0.05 (Welch's T-test). Values in bold indicate the fold change for each metabolite (WT vs pykAT>D). Fig. S4. LC/MS analysis of legionaminic acid in the metabolome. A. Extracted ion chromatogram (EIC) corresponds to the deprotonated molecule [M-H]- at m/z 333.1303 (5 ppm accuracy). B. Zoom on the mass spectrum of legionaminic acid in the negative mode. C. Collision Induced dissociation (CID) spectrum of legionaminic acid in the negative mode at 22% Normalized Collision Energy (NCE). D. CID spectrum of legionaminic acid in the p [file 12915_2022_1278_MOESM1_ESM.zip › Fig. S6.pptx]

## Slide 1
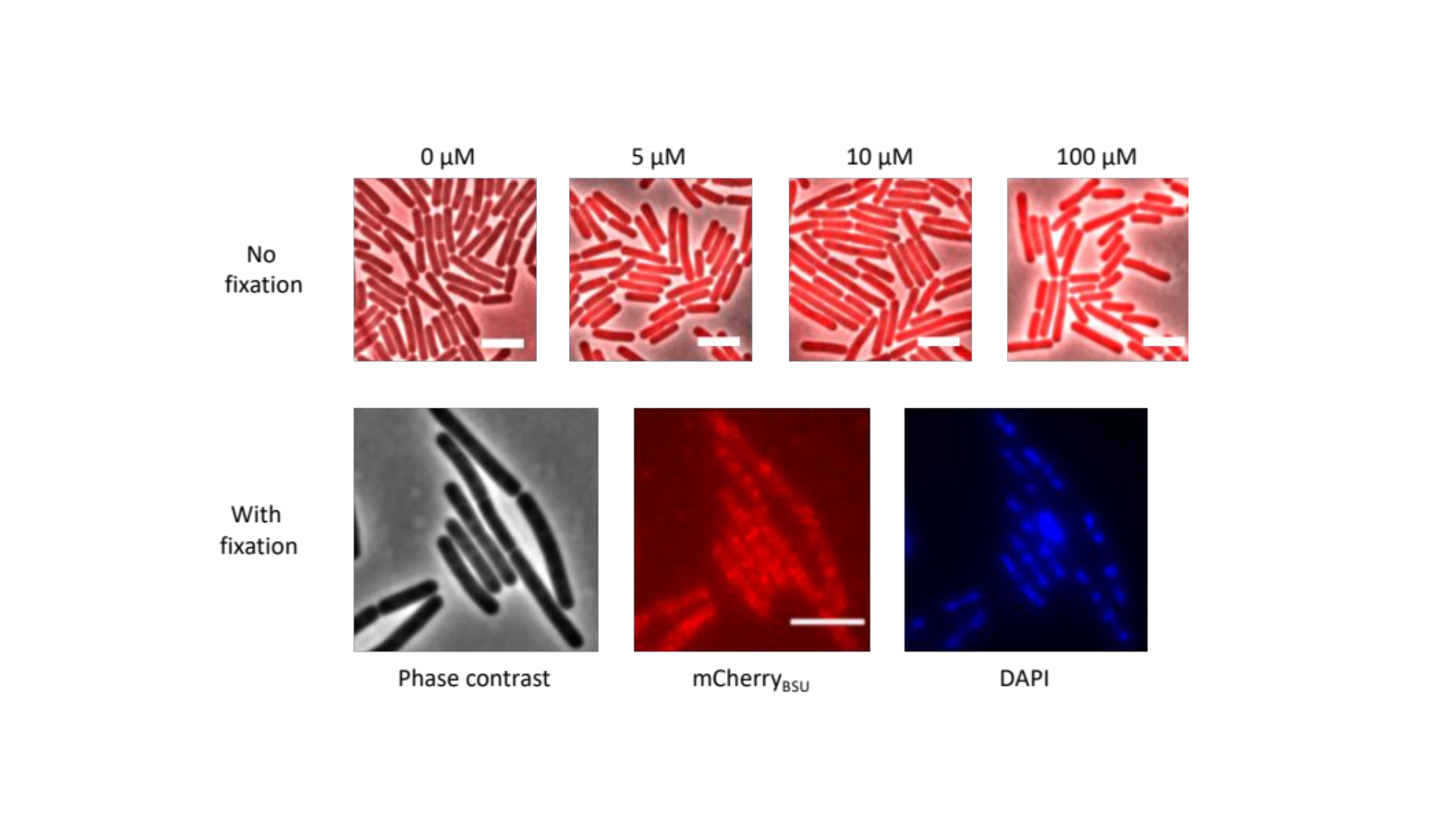

Supplement: Supplementary file 1 — Additional file 1: Fig. S1. Key amino-acids of the Cat and PEPut domains of PykA. A. Cat domain analysis. Clustalw and Chimera analysis of the pyruvate kinase of B. subtilis (PykA), human cells (PKM2) and Mycobacterium tuberculosis (PYK) identified key amino acids of the catalytic site of the B. subtilis protein. B. PEPut domain analysis. Alignment of the PEPut domain of PykA to related domains of various metabolic enzymes. The red arrow highlights the conserved LTSH motif (coordinates 536-539). Fig. S2. Effect of Cat and PEPut mutations on growth in MC. Wild-type and pykA mutants were first grown over-night in MC supplemented with antibiotic when appropriate. Upon saturation, cultures were diluted 1000-fold in the same medium without antibiotic and growth was monitored spectrophometrically. Left panel: Analysis of catalytic mutants (pykAΔcat, pykAR32A, pykAR73A, pykAK220A, pykAGD245/6AA, pykAT278A, pykAJP). Right panel: Analysis of PEPut and Cat-PEPut interaction mutants (pykAΔPEP, pykAT>A, pykAS>A, pykAH>A, pykATSH>AAA, pykAT>D, pykAS>D, pykAH>D, pykATSH>DDD, pykAE209A, pykAL536A). Controls: TF8A (wild-type) and ΔpykA. Fig. S3. Analysis of NTP in the metabolome of wild-type and pykAT>D cells. ATP, GTP and CTP were detected in the positive ionization mode. UTP was detected in the negative ionization mode. Note that TTP signals were too low for quantifications. Data correspond to 3 independent extractions (solid cultures).*, p > 0.05 ; **, p < 0.05 (Welch's T-test). Values in bold indicate the fold change for each metabolite (WT vs pykAT>D). Fig. S4. LC/MS analysis of legionaminic acid in the metabolome. A. Extracted ion chromatogram (EIC) corresponds to the deprotonated molecule [M-H]- at m/z 333.1303 (5 ppm accuracy). B. Zoom on the mass spectrum of legionaminic acid in the negative mode. C. Collision Induced dissociation (CID) spectrum of legionaminic acid in the negative mode at 22% Normalized Collision Energy (NCE). D. CID spectrum of legionaminic acid in the p [file 12915_2022_1278_MOESM1_ESM.zip › Fig. S7.pptx]

## Slide 1
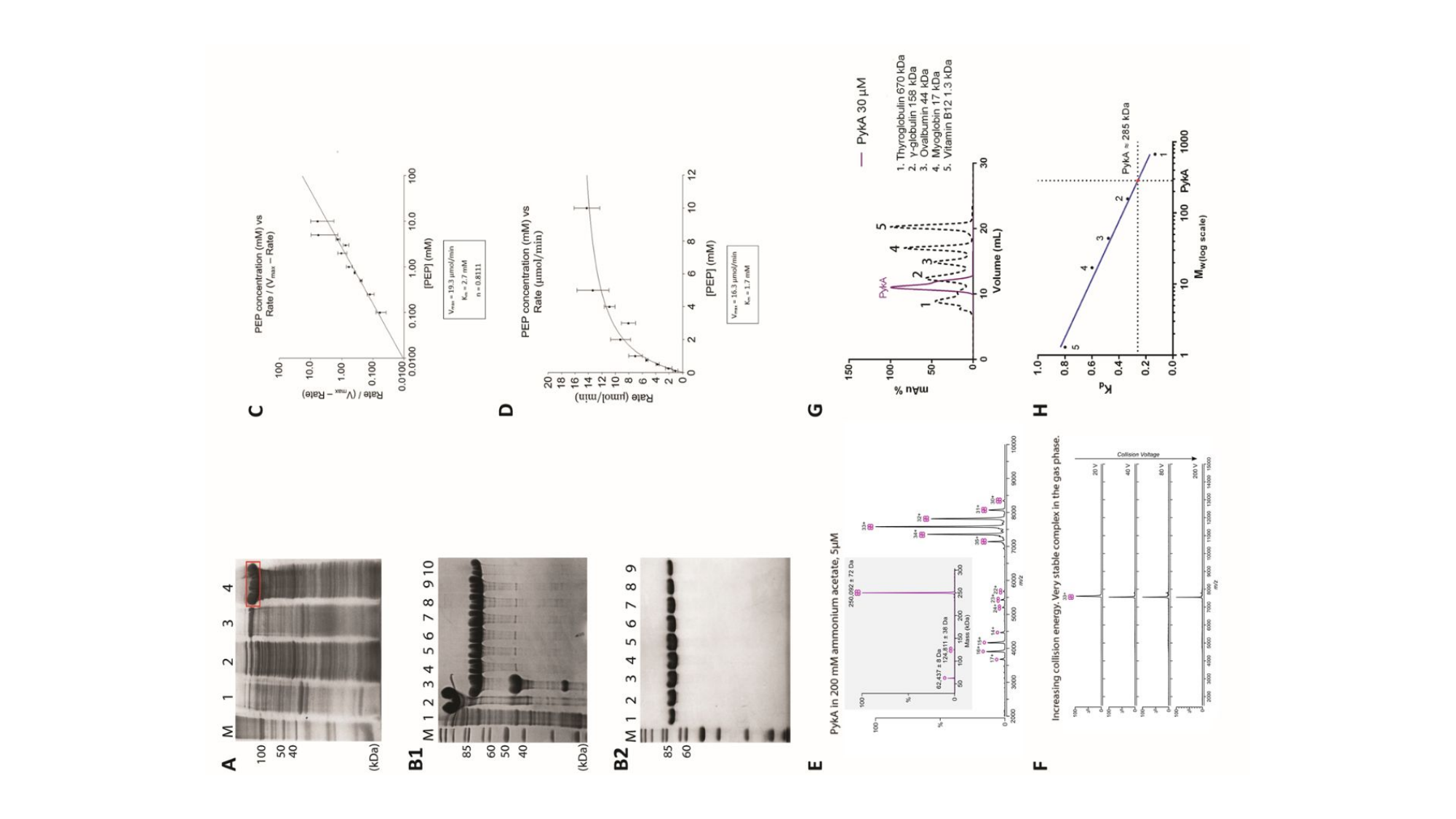

Supplement: Supplementary file 1 — Additional file 1: Fig. S1. Key amino-acids of the Cat and PEPut domains of PykA. A. Cat domain analysis. Clustalw and Chimera analysis of the pyruvate kinase of B. subtilis (PykA), human cells (PKM2) and Mycobacterium tuberculosis (PYK) identified key amino acids of the catalytic site of the B. subtilis protein. B. PEPut domain analysis. Alignment of the PEPut domain of PykA to related domains of various metabolic enzymes. The red arrow highlights the conserved LTSH motif (coordinates 536-539). Fig. S2. Effect of Cat and PEPut mutations on growth in MC. Wild-type and pykA mutants were first grown over-night in MC supplemented with antibiotic when appropriate. Upon saturation, cultures were diluted 1000-fold in the same medium without antibiotic and growth was monitored spectrophometrically. Left panel: Analysis of catalytic mutants (pykAΔcat, pykAR32A, pykAR73A, pykAK220A, pykAGD245/6AA, pykAT278A, pykAJP). Right panel: Analysis of PEPut and Cat-PEPut interaction mutants (pykAΔPEP, pykAT>A, pykAS>A, pykAH>A, pykATSH>AAA, pykAT>D, pykAS>D, pykAH>D, pykATSH>DDD, pykAE209A, pykAL536A). Controls: TF8A (wild-type) and ΔpykA. Fig. S3. Analysis of NTP in the metabolome of wild-type and pykAT>D cells. ATP, GTP and CTP were detected in the positive ionization mode. UTP was detected in the negative ionization mode. Note that TTP signals were too low for quantifications. Data correspond to 3 independent extractions (solid cultures).*, p > 0.05 ; **, p < 0.05 (Welch's T-test). Values in bold indicate the fold change for each metabolite (WT vs pykAT>D). Fig. S4. LC/MS analysis of legionaminic acid in the metabolome. A. Extracted ion chromatogram (EIC) corresponds to the deprotonated molecule [M-H]- at m/z 333.1303 (5 ppm accuracy). B. Zoom on the mass spectrum of legionaminic acid in the negative mode. C. Collision Induced dissociation (CID) spectrum of legionaminic acid in the negative mode at 22% Normalized Collision Energy (NCE). D. CID spectrum of legionaminic acid in the p [file 12915_2022_1278_MOESM1_ESM.zip › Fig. S8.pptx]

## Slide 1
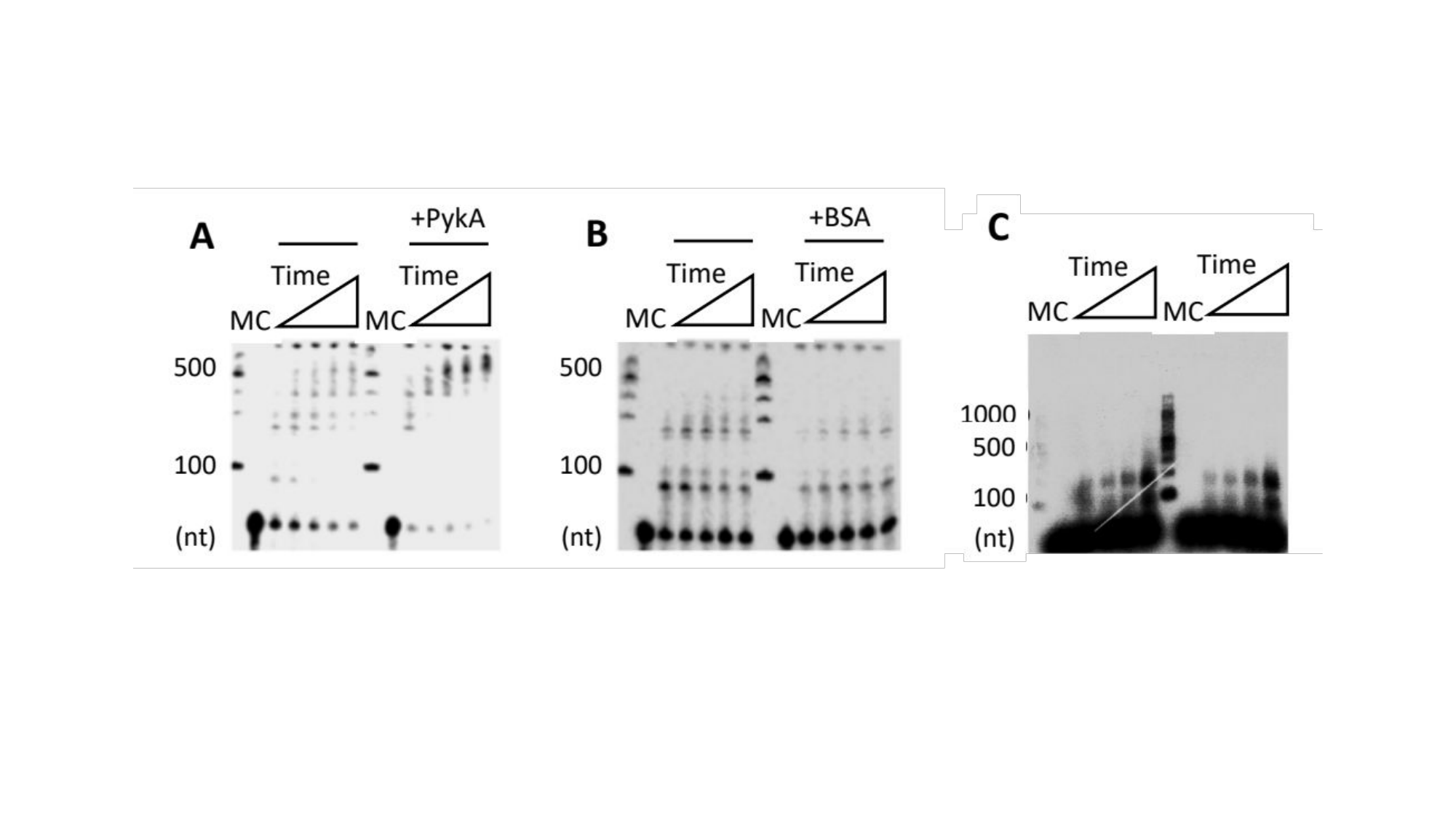

Supplement: Supplementary file 1 — Additional file 1: Fig. S1. Key amino-acids of the Cat and PEPut domains of PykA. A. Cat domain analysis. Clustalw and Chimera analysis of the pyruvate kinase of B. subtilis (PykA), human cells (PKM2) and Mycobacterium tuberculosis (PYK) identified key amino acids of the catalytic site of the B. subtilis protein. B. PEPut domain analysis. Alignment of the PEPut domain of PykA to related domains of various metabolic enzymes. The red arrow highlights the conserved LTSH motif (coordinates 536-539). Fig. S2. Effect of Cat and PEPut mutations on growth in MC. Wild-type and pykA mutants were first grown over-night in MC supplemented with antibiotic when appropriate. Upon saturation, cultures were diluted 1000-fold in the same medium without antibiotic and growth was monitored spectrophometrically. Left panel: Analysis of catalytic mutants (pykAΔcat, pykAR32A, pykAR73A, pykAK220A, pykAGD245/6AA, pykAT278A, pykAJP). Right panel: Analysis of PEPut and Cat-PEPut interaction mutants (pykAΔPEP, pykAT>A, pykAS>A, pykAH>A, pykATSH>AAA, pykAT>D, pykAS>D, pykAH>D, pykATSH>DDD, pykAE209A, pykAL536A). Controls: TF8A (wild-type) and ΔpykA. Fig. S3. Analysis of NTP in the metabolome of wild-type and pykAT>D cells. ATP, GTP and CTP were detected in the positive ionization mode. UTP was detected in the negative ionization mode. Note that TTP signals were too low for quantifications. Data correspond to 3 independent extractions (solid cultures).*, p > 0.05 ; **, p < 0.05 (Welch's T-test). Values in bold indicate the fold change for each metabolite (WT vs pykAT>D). Fig. S4. LC/MS analysis of legionaminic acid in the metabolome. A. Extracted ion chromatogram (EIC) corresponds to the deprotonated molecule [M-H]- at m/z 333.1303 (5 ppm accuracy). B. Zoom on the mass spectrum of legionaminic acid in the negative mode. C. Collision Induced dissociation (CID) spectrum of legionaminic acid in the negative mode at 22% Normalized Collision Energy (NCE). D. CID spectrum of legionaminic acid in the p [file 12915_2022_1278_MOESM1_ESM.zip › Fig. S9.pptx]
